# From isolation to insights: mitochondrial complex I in the diatom *Phaeodactylum tricornutum*

**DOI:** 10.1111/tpj.70706

**Published:** 2026-01-29

**Authors:** Federico Berdun, Jennifer Senkler, Michael Senkler, Noah Ditz, Eva Plönnigs, Thomas Reinard, Eduardo Zabaleta, Hans‐Peter Braun

**Affiliations:** ^1^ Instituto de Investigaciones Biológicas IIB‐CONICET Universidad Nacional de Mar del Plata Mar del Plata Argentina; ^2^ Faculty of Natural Sciences Institute of Plant Genetics, Leibniz Universität Hannover Hannover Germany

**Keywords:** diatoms, *Phaeodactylum tricornutum*, mitochondria, respiration, respiratory chain, complex I, NADH dehydrogenase, carbonic anhydrase, carbonic anhydrase module, ferredoxin bridge

## Abstract

Diatoms are among the most ecologically successful microalgae, contributing significantly to marine primary production and global carbon cycling. Their distinctive metabolic architecture, shaped by a complex evolutionary history involving secondary endosymbiosis, includes a highly compartmentalized cell organization and unique metabolic pathways. In *Phaeodactylum tricornutum*, a model pennate diatom, chloroplasts with four membranes and mitochondria of likely exosymbiotic origin exhibit intricate physical and metabolic interactions that support integrated carbon and nitrogen metabolism. The mitochondrial electron transport chain, essential for ATP synthesis, shows clade‐specific structural and compositional adaptations. Despite its importance, detailed proteomic characterization has remained limited. Here, we report a method for the isolation of mitochondrial complex I from *P. tricornutum* and present a comprehensive proteomic analysis. Our results confirm the presence of carbonic anhydrase and bridge modules, both previously proposed as ancestral features of mitochondrial complex I, and identify at least one novel, clade‐specific subunit that resembles NAD(P)H‐dependent trans‐2‐enoyl‐CoA/ACP reductases (TER) from other species. The subunit is similar to proteins involved in mitochondrial fatty acid biosynthesis. Our findings provide new insights into the composition, evolutionary conservation, and potential biotechnological relevance of this essential respiratory protein complex in diatoms.

## Significance Statement

Diatoms are of great ecological importance on a global scale, but their photosynthesis and, in particular, their respiration are only partially understood. Here, we report a protocol for the purification of mitochondrial complex I of the respiratory chain in the model diatom *Phaeodactylum tricornutum* and a proteomic analysis of its composition, which provides insights into the function and evolution of this respiratory enzyme complex.

## INTRODUCTION

Diatoms are free‐living unicellular photosynthetic microorganisms and the most abundant group present in the phytoplankton. They contribute around 40% of global CO_2_ fixation in the oceans and play a central role in the trophic network as primary producers, being one of the most ecologically successful groups of microalgae worldwide (Falciatore & Mock, 2022; Wilhelm et al., 2006).

Diatoms are exceptionally well adapted to their environment, largely due to a unique metabolism shaped by their complex evolutionary history involving secondary endosymbiosis (Benoiston et al., 2017). Their chloroplasts are surrounded by four membranes: the two outermost, known as (i) the chloroplast endoplasmic reticulum membrane (cERM), which also is interconnected with the mitochondrial network (Uwizeye et al., 2021); and (ii) the periplastidial membrane (PPM); and finally, the two innermost membranes, which correspond to (iii) the outer and (iv) the inner envelope membranes of the chloroplast (oEM and iEM) (Flori et al., 2016, 2017). This intricate membrane architecture reflects their chimeric origin and enables unique metabolic interactions. Among these, the ornithine‐urea cycle (OUC), which resembles the one found in metazoans, plays a key role and is tightly linked to the tricarboxylic acid (TCA) cycle. This connection enables diatoms to efficiently coordinate carbon and nitrogen metabolism in response to environmental demands (Allen et al., 2011).

Another feature contributing to their ecological success is the level of C4‐like photosynthetic enzymes, as found in *Phaeodactylum tricornutum*, a pennate diatom widely used as a model organism. Enzymes such as phosphoenolpyruvate carboxylase (PEPC), pyruvate orthophosphate dikinase (PPDK), and decarboxylases allow for efficient inorganic carbon utilization, even under low CO_2_ (Haimovich‐Dayan et al., 2013). Unlike the situation in C4 plants, these enzymes are localized in unconventional compartments, such as mitochondria and the periplastidial space, rather than in the cytosol.

Several studies have highlighted the intricate interactions between chloroplasts, with their additional membranes, and mitochondria (of supposed exosymbiotic origin) in diatoms. These interactions involve not only extensive physical contact, which may vary depending on environmental conditions, but also an intensive metabolic exchange that is likely of physiological relevance (Bailleul et al., 2015; Uwizeye et al., 2017, 2021). For *P. tricornutum*, it was shown that the mitochondria represent a reticular continuum that is surrounding a large central chloroplast. In some other diatoms, the mitochondria are roundish to elongated compartments, for example, in *Thalassiosira pseudonana*. Mitochondria‐enriched fractions were successfully prepared from this species (Schober et al., 2019). Proteomic analysis revealed 49 mitochondrial proteins, including 27 proteins that form part of the five protein complexes of the oxidative phosphorylation (OXPHOS) system. This system, comprising complexes of the respiratory electron transfer chain (complexes I–IV) and the mobile electron carriers ubiquinone and cytochrome *c*, transports electrons from the reduced coenzymes NADH + H^+^ and FADH_2_ to molecular oxygen, the final electron acceptor, while simultaneously translocating protons across the inner mitochondrial membrane. The resulting proton‐motive force drives ATP synthase (complex V) to phosphorylate ADP, thereby generating ATP (Braun, 2020; Meyer et al., 2022; Møller et al., 2021).

In most eukaryotic organisms, the mitochondrial OXPHOS system consists of far more than 100 different proteins; Complex I of the OXPHOS system alone consists of 43 subunits in *Yarrowia lipolytica* (Parey et al., 2019), 45 in mammals (Kampjut & Sazanov, 2020), 48 in Arabidopsis, and 51 in Polytomella (Klusch et al., 2021). In mitochondria of protozoans, complex I is even larger (Han et al., 2023; He et al., 2024; Mühleip et al., 2023; Shin et al., 2025; Zhou et al., 2022). None of the OXPHOS complexes of any diatom species has ever been purified and characterized in detail. We report here on the first purification of complex I from a diatom.

Complex I is a NADH:ubiquinone oxidoreductase, which significantly contributes to the formation of the proton gradient across the inner mitochondrial membrane (Kühlbrandt et al., 2025). It is the main entry point for electrons into the mitochondrial respiratory chain. The complex consists of two elongated domains called arms: a membrane arm that inserts largely into the inner mitochondrial membrane and a peripheral arm that extends far into the mitochondrial matrix. Together the two arms form an L‐like structure. Both arms are subdivided into two functional modules: the peripheral arm into the NADH‐dehydrogenase module (N module) and the ubiquinone reduction module (Q module), and the membrane arm, which is responsible for proton translocation (P) across the inner mitochondrial membrane, into a domain located proximal to the peripheral arm (P_P_ module) and a domain located distal to the peripheral arm (P_D_ module).

In protists, algae, and plants, a carbonic anhydrase module is attached to the membrane arm on its matrix‐exposed side. This module is composed of trimers of gamma‐type carbonic anhydrases (CAs). It might be involved in delivering protons to the membrane arm of complex I for subsequent translocation across the inner mitochondrial membrane (Braun & Klusch, 2024). Analyses of complex I from Arabidopsis and Polytomella by single particle cryogenic electron microscopy (CryoEM) revealed that the carbonic anhydrase module is connected via three subunits to the peripheral arm (Klusch et al., 2021). This connection is designated the bridge module and includes an unusual ferredoxin. The presence of the bridge module has also been shown in *Tetrahymena* (Han et al., 2023; Mühleip et al., 2023; Zhou et al., 2022), and Euglena (He et al., 2024; Maldonado, 2024). In contrast, the carbonic anhydrase and bridge modules are absent in animals and fungi. The presence of a CA module integrated into complex I is considered an ancestral trait. Previous work identified subunits of this module in *P. tricornutum*, reinforcing the ancestral nature of its electron transport chain (Cainzos et al., 2021).

We report here the purification of complex I from the model diatom *P. tricornutum*. A fraction enriched in organellar membranes is prepared by a combination of French press treatment, mortar treatment, and various centrifugation steps. The membranes are then solubilized using mild detergent and protein complexes separated by means of sucrose gradient ultracentrifugation. Being the largest protein complex of our membrane fraction, complex I is separated nicely from other complexes. Proteomic analyses enabled identification of over 30 proteins homologous to complex I subunits of other organisms, as well as at least one additional subunit specific to *P. tricornutum*. Resulting insights into the evolution of complex I are discussed.

## RESULTS

### Enrichment of subcellular compartments from *Phaeodactylum tricornutum*

*Phaeodactylum tricornutum* has been shown to include a single large chloroplast, which is surrounded by a reticular and continuous mitochondrial network (Uwizeye et al., 2021). A classical mitochondrial isolation method, as established for seed plants, in which mitochondria have a shape similar to that of bacteria, is therefore not possible. To characterize the mitochondria from *P. tricornutum*, the rigid silica cell wall must first be opened using mechanical force. During this process, both the chloroplast and the associated mitochondrial network can be easily destroyed. Fragmentation of the chloroplast and the mitochondrial network may result in the formation of vesicles derived from either origin. Using differential and density gradient centrifugation, we therefore attempted to generate subcellular fractions in which mitochondrial and chloroplast proteins are enriched. First, however, we optimized the cell disruption process. In line with reports in the literature, we tested French press treatment, mortar treatment, and a combination of both methods.

**Figure 1:**
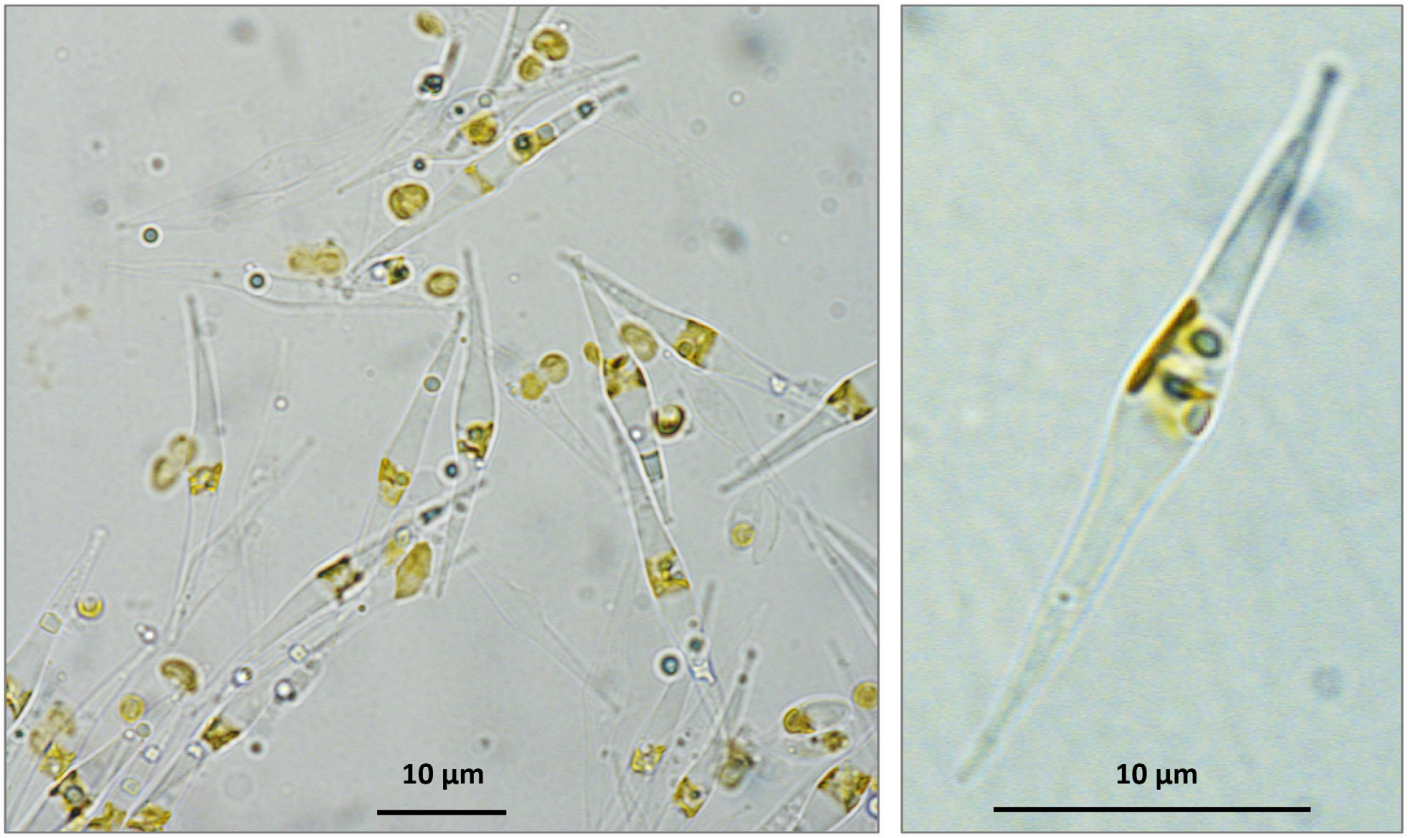
Microscopic images of *Phaeodactylum tricornutum* culture. Freshly cultured cells (SAG strain 1090‐6, stationary phase) were analyzed using a light microscope at 100× resolution. Scale bars = 10 μm.

**Figure 2:**
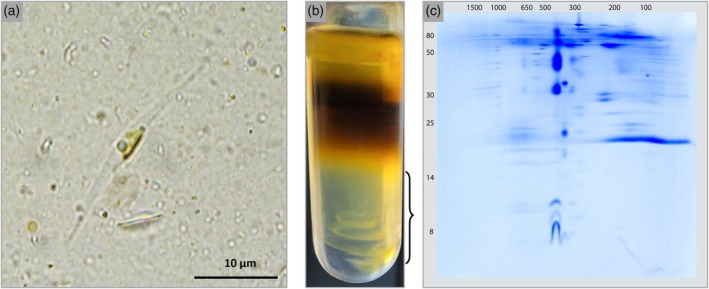
Cell disruption and organelle isolation. (a) Light microscope image of *P. tricornutum* cells following cell disruption treatment (100× resolution, scale bar = 10 μm). Besides one cell that remained intact (center), the image reveals numerous subcellular fragments. (b) Separation of a *P. tricornutum* fraction on a continuous percoll gradient. The bracket (right) indicates the organelle‐enriched fraction that was utilized for further analyses. (c) Analysis of the organelle‐enriched fraction by 2D Blue native (BN)/SDS PAGE. Separated proteins were visualized by Coomassie staining. The molecular masses of standard proteins are given at the top (protein complexes, BN PAGE) and to the left (protein subunits, SDS PAGE) of the 2D gel in kDa.

*P. tricornutum* cells were cultivated as described in the ‘Materials and methods’ section. The state of the cultures was observed microscopically (Figure 1). Cells were harvested via centrifugation. For cell disruption, we tested various treatments (French press treatment with high pressure [138 MPa]; with low pressure [20 MPa]; two successive French press treatments; French press treatment followed by mortar treatment; mortar treatment alone). The effectiveness of each method was evaluated microscopically. The aim was to identify conditions that disrupted the majority of cells and simultaneously led to the generation of subcellular particles. Subcellular particles were enriched using centrifugations and density gradient centrifugations under different conditions and subsequently analyzed by 2D Blue native/SDS polyacrylamide gel electrophoresis (2D BN/SDS PAGE) with regard to the presence of protein complexes (Figure S1). Based on a comparison of the obtained 2D gels with reference gels for *Arabidopsis thaliana* (Behrens et al., 2013; Klodmann & Braun, 2011), it became apparent that our fractions contained predominantly thylakoid membrane complexes, especially photosystem II and the *b*_6_ *f* complex. When low pressures French press treatment was used, the RubisCO complex from the chloroplast stroma was also visible, indicating that chloroplasts stayed (at least partly) intact (Figure S1d). A particularly large protein complex in the 1000 kDa area, composed of a large number of different subunits, was assumed to be mitochondrial complex I (as confirmed in the following). This complex served as a marker to trace a relative enrichment of mitochondrial membranes (Figure S1f). Our strategy to optimize cell disruption and organelle isolation for *P. tricornutum* is illustrated in Figure 2; the final protocol for purifying organelle membranes from *P. tricornutum* is described in detail in the ‘Materials and methods’ section and illustrated in Figure S2. Chloroplast protein complexes predominate in this fraction. We refer to this fraction in the following as the ‘organelle‐enriched fraction’.

### Proteomic characterization of the organelle‐enriched fraction from *P. tricornutum*

**Figure 3:**
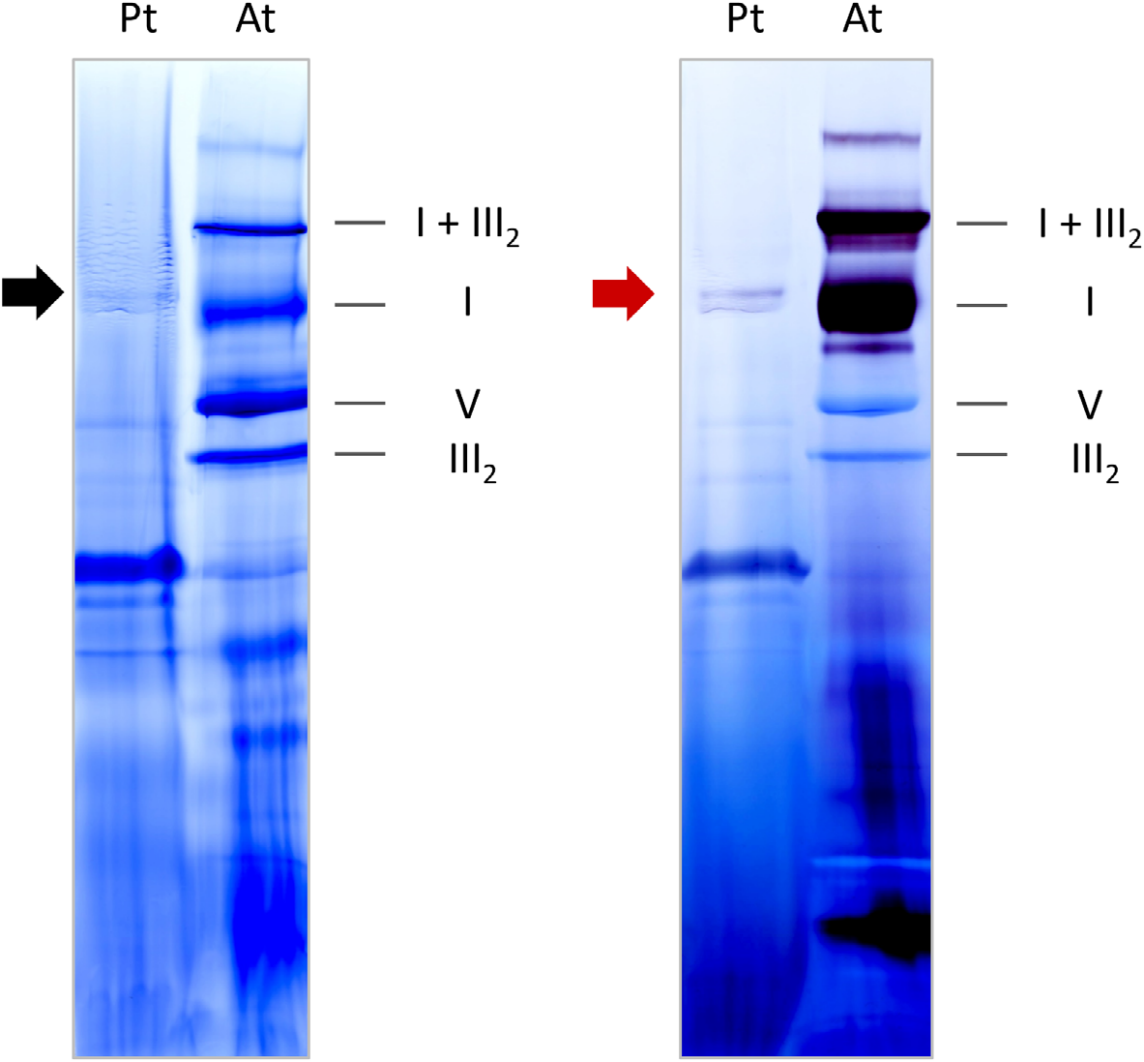
*In‐gel* activity staining of complex I. Protein complexes of an organelle‐enriched fraction of *Phaeodactylum tricornutum* (Pt) and a purified mitochondrial fraction of *Arabidopsis thaliana* (At) were separated by 1D BN PAGE. The gels were either Coomassie‐stained (left image) or by NADH‐dehydrogenase (complex I) activity staining (right image). The identities of OXPHOS complexes from Arabidopsis are given to the right of the gel images. I + III_2_: supercomplex composed of monomeric complex I and dimeric complex III (1500 kDa); I: monomeric complex I (1000 kDa), V: ATP synthase complex (650 kDa); III_2_: dimeric complex III (500 kDa). The black arrow (left) indicates putative *P. tricornutum* complex I; the red arrow (right) indicates a *P. tricornutum* complex exhibiting NADH‐dehydrogenase (complex I) activity.

For further characterization, the organelle‐enriched fraction was separated by 1D BN PAGE and the resulting gel was either stained with Coomassie blue or by an *in‐gel* assay for NADH‐dehydrogenase (complex I) activity (Figure 3). For comparison, a purified mitochondrial fraction from *Arabidopsis thaliana* was analyzed in parallel. The known mitochondrial protein complexes from *A. thaliana* were barely visible in *P. tricornutum*, with the exception of a weak band running slightly above the complex I band from *A. thaliana*. Based on the known molecular mass of complex I from *A. thaliana*, which is 1000 kDa (Klusch et al., 2023), the molecular mass of the putative *P. tricornutum* complex I can be estimated to be approximately 1050 kDa (Figure 3, left). In fact, this protein complex showed *in‐gel* NADH‐dehydrogenase activity (Figure 3, right).

**Figure 4:**
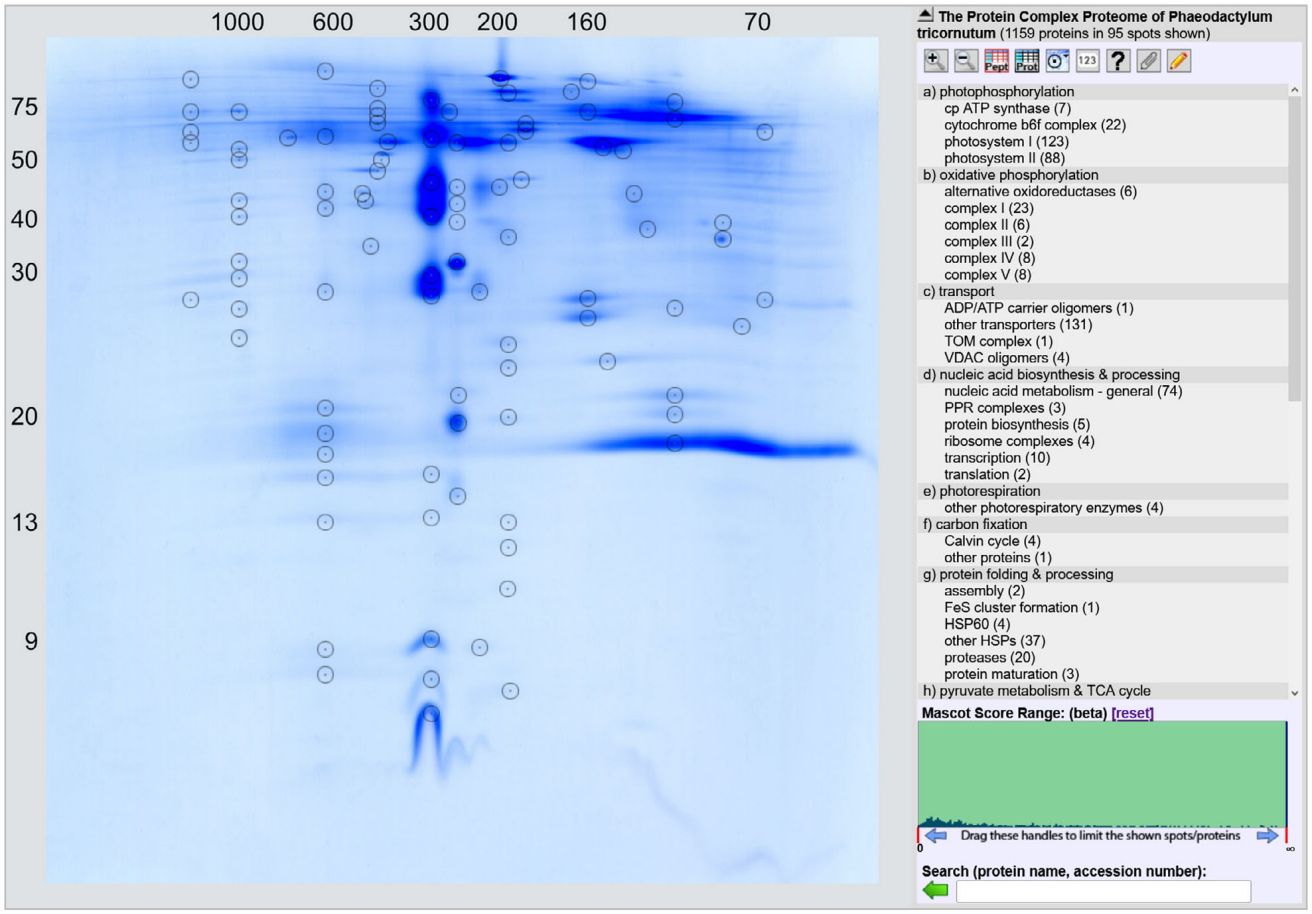
Reference map of the ‘Protein Complex Proteome’ of *P. tricornutum*. The proteome of an organelle‐enriched fraction isolated from *P. tricornutum* was separated by 2D BN/SDS PAGE and the resulting gel was uploaded onto the GelMap portal (https://gelmap.de/). Proteins from 95 spots (black circles) were identified by mass spectrometry. Results are directly accessible at https://www.gelmap.de/phaeo‐organelles/ by clicking the spots (for a detailed manual of GelMap see Senkler & Braun, 2012). Proteins are assigned to protein complexes and functional categories (table to the right). Numbers above the gel indicate molecular masses of standard protein complexes (first gel dimension); numbers left of the gel are molecular masses of standard proteins in kDa.

For further characterization of the organelle‐enriched fraction, 2D BN‐SDS PAGE was performed, and the resulting 2D gel was stained with Coomassie blue (Figure 4). Visible protein spots were cut out and the contained proteins were identified by mass spectrometry. The results were evaluated using a *P. tricornutum* protein database (downloaded from UniProt; www.uniprot.org; Proteome ID UP000000759). A total of 95 protein spots were analyzed containing a cumulated total of 1159 proteins. The identities of the proteins are listed in Table S1. Furthermore, a GelMap was generated and is accessible at our GelMap portal at https://www.gelmap.de/phaeo‐organelles/. On the GelMap, identities of proteins are displayed upon clicking on the individual gel spots.

Numerous protein complexes were identified, including a particularly abundant protein complex at 280 kDa, which is the core complex of photosystem II (the D1, D2, CP43, CP47 proteins, the α and β subunits of cytochrome *b*559 and further photosystem II subunits), and the chloroplast cytochrome *b*_6_ *f* complex at 260 kDa (cytochrome *f*, cytochrome *b*_6_, the Rieske FeS protein, subunit IV). Subunits of photosystem I form a complex of about 600 kDa (the large core subunits A and B, subunits D, E, F, and L and several further small photosystem I subunits), together with fucoxanthin‐binding proteins. Only a few mitochondrial proteins were identified. However, it was found that the 1050 kDa protein complex is indeed complex I. A total of eight different complex I subunits were identified: the 75 kDa, 51 kDa, 39 kDa, B17.2 and the ND7 subunits of the peripheral arm of complex I, the ND5 and ND2 subunits of the membrane arm and at least one subunit of the carbonic anhydrase module (Figure 4; Table S1, GelMap at https://www.gelmap.de/phaeo‐organelles/).

To estimate the composition of our organelle‐enriched fraction, the 1159 identified proteins were assigned to subcellular compartments based on their homologous Arabidopsis proteins (specified by the Subcellular localization database for Arabidopsis proteins [SUBA], Version 5, https://suba.live/) (Hooper et al., 2014). iBAQ values of the 1159 proteins identified by MS were subsequently summed up per subcellular fraction (Figure S3). The results show that approximately 66% of the protein abundance originated from chloroplasts and approximately 4% from mitochondria. It hence can be estimated that our organelle‐enriched fraction contains close to 70% chloroplast or mitochondrial proteins.

### Proteomic characterization of a complex I‐enriched fraction

**Figure 5:**
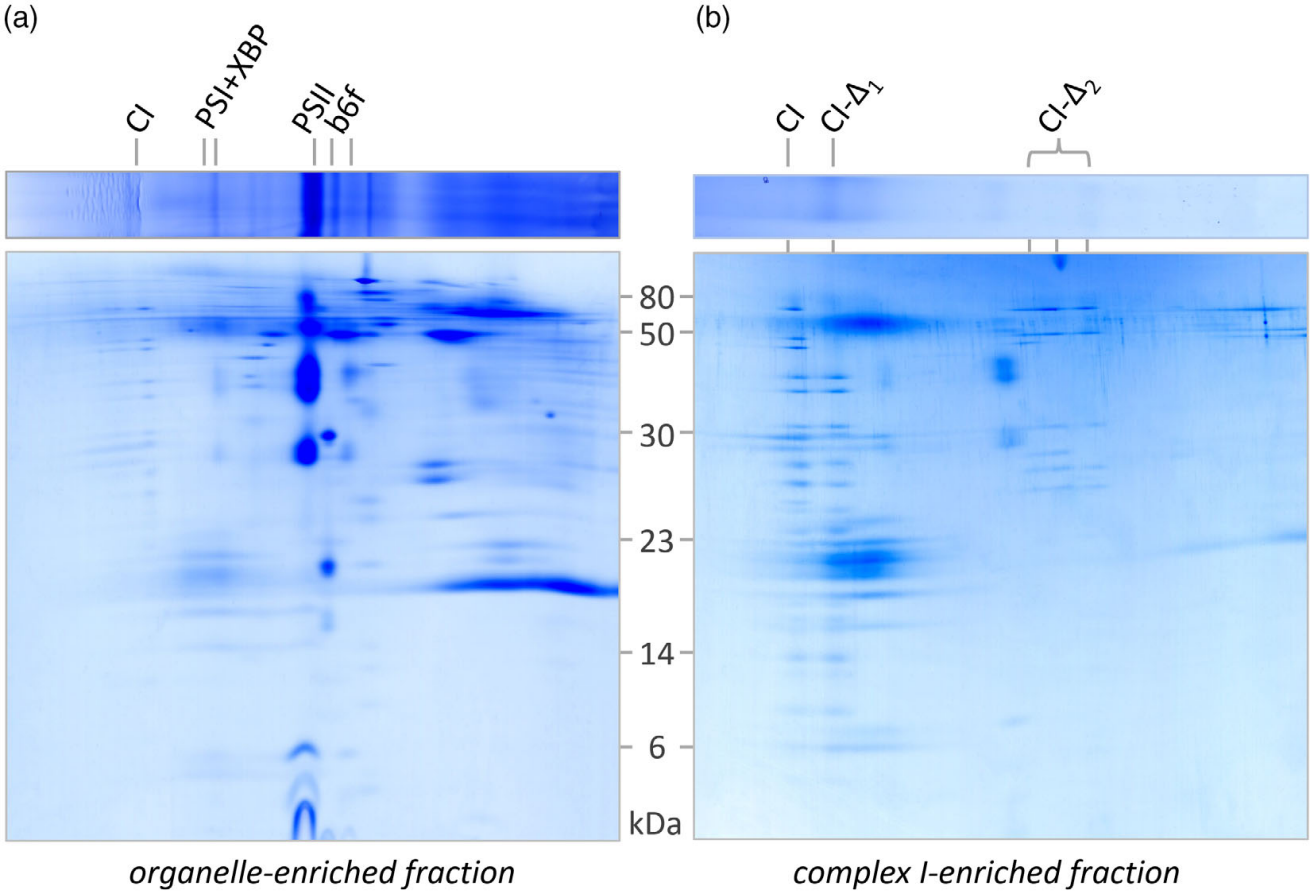
Comparison of organelle‐ and complex I‐enriched fractions from *P. tricornutum* by 2D BN/SDS PAGE. (a) 2D BN‐SDS gel of an organelle‐enriched fraction of *P. tricornutum*. (b) 2D BN‐SDS gel of a complex I‐enriched fraction of *P. tricornutum*. Molecular masses of standard proteins are indicated between the two gels (in kDa). Identities of protein complexes are given on top of the two gels. CI = complex I (estimated molecular mass: 1050 kDa); PSI + XBP = PSI + fucoxanthin‐binding proteins (~600 kDa); PSII = photosystem II core complex (~ 280 kDa); *b*_6_ *f* = *b*_6_ *f* complex (~260 kDa); CI‐Δ1 and CI‐Δ2 = subcomplexes of complex I (~850 and ~200 kDa).

Since complex I in our organelle‐enriched fraction is the largest visible protein complex, we decided to further purify this complex by means of a size separation. Sucrose ultracentrifugation was used for this purpose (details are given in the “Materials and methods” section and illustrated in Figure S4). After ultracentrifugation, the resulting sucrose gradient was fractionated and each fraction was analyzed individually by 1D BN PAGE (Figure S5). One fraction, which contained a particularly distinct complex I band, was subsequently analyzed by 2D BN/SDS PAGE. Complex I was found to be considerably enriched compared to the organelle‐enriched fraction (Figure 5). Several additional subunits are visible. Compared to the organelle‐enriched fraction, the amount of photosystem II is dramatically reduced while photosystem I with its fucoxanthin‐binding proteins is less reduced, as the molecular mass of this complex is closer to that of complex I. We refer to this fraction in the following as the ‘complex I‐enriched fraction’. Besides the 1050 kDa complex I from *P. tricornutum*, this fraction also includes smaller versions of this protein complex, running at about 850, as well as 230, 200, and 170 kDa. All these complexes lack distinct subunits of the 1050 kDa holo‐complex. We conclude that complex I from *P. tricornutum* partially dissociates into subcomplexes during the last purification step. Still, about 50% of complex I remained intact.

**Figure 6:**
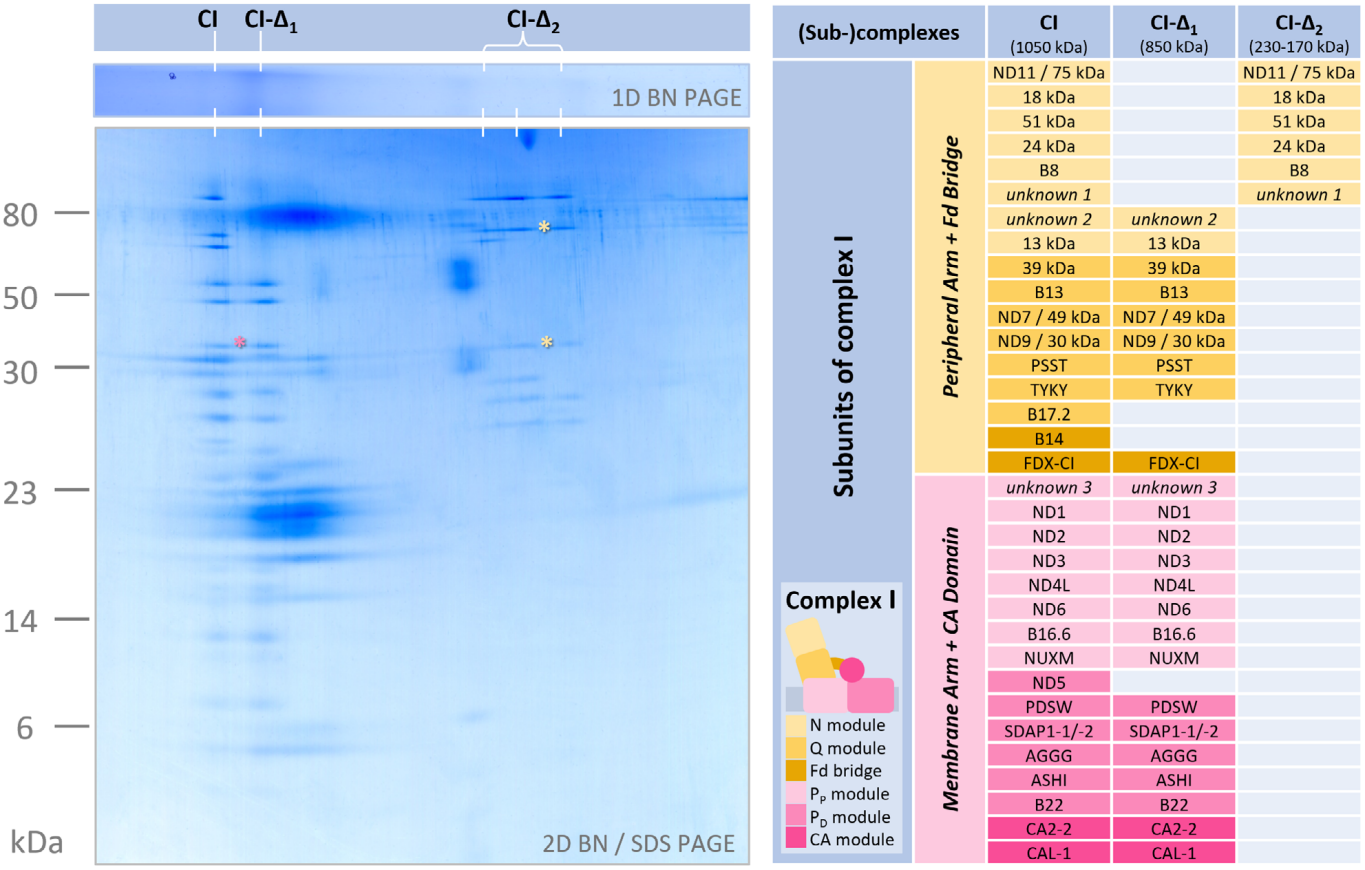
Proteomic analysis of a complex I‐enriched fraction from *P. tricornutum*. The fraction was separated by 2D BN/SDS PAGE. Molecular masses of standard proteins are given to the left of the gel in kDa. Intact complex I (CI) runs on the BN gel dimension at about 1050 kDa; complex I dissection products (Δ1, Δ2) run at about 850 and 170–230 kDa. Complex I subunits were analyzed by mass spectrometry and identified based on homology searches. A reference map of all identified *P. tricornutum* complex I subunits is available at https://www.gelmap.de/phaeo‐ci; see Figure S6. The subunit composition of *P. tricornutum* complex I and the compositions of the complex I dissection products are given in the table to the right of the gel image. Proteins are sorted according to their occurrence in complex I modules as known for complex I of *Arabidopsis thaliana* (Klusch et al., 2021, 2023); see color scheme within the table. Fd bridge = ferredoxin bridge module; N module = NADH oxidation module; P_D_ module = proton translocation module, distal to the peripheral arm; P_P_ module = proton translocation module, proximal to the peripheral arm; Q module = quinone reduction module. Asterisks on the gel indicate putative *P. tricornutum* complex I subunits not present in other clades of species.

We used a 2D BN/SDS gel of our complex I‐enriched fraction to systematically identify complex I subunits of *P. tricornutum* (Figure 6). Our complex I dataset can be accessed via another GelMap (Figure S6, GelMap at https://www.gelmap.de/phaeo‐ci/). We identified 15 subunits homologous to proteins of the peripheral arm of complex I from other clades of eukaryotes, 6 of which form part of the N module, 7 of the Q module, and 2 of the bridge module, including the recently described complex I‐integrated ferredoxin subunit (Klusch et al., 2021). Furthermore, 15 subunits homologous to proteins of the membrane arm of complex I from other clades of eukaryotes were identified, 7 of which belong to the P_P_ module, 6 to the P_D_ module, and 2 to the carbonic anhydrase module.

**Figure 7:**
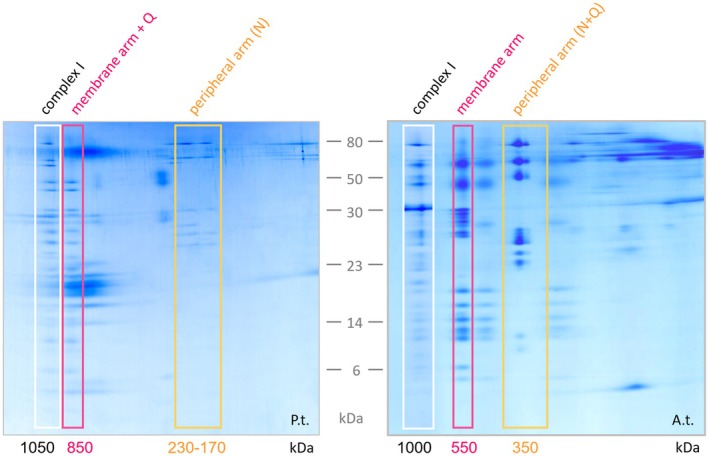
Comparison of complex I subcomplexes of *P. tricornutum* and *A. thaliana*. *Image to the left*: 2D BN/SDS gel of the complex I‐enriched fraction of *P. tricornutum* (P.t.). The 850 kDa dissection product includes the complete membrane arm and additionally the Q module of the peripheral arm. The 230–170 kDa dissection products include the subunits of the N module. *Image to the right*: 2D BN/SDS gel of SDS‐induced dissection products of *A. thaliana* complex I (A.t.), taken from (Klodmann et al., 2010). The 550 kDa dissection product includes the subunits of the membrane arm; the 350 kDa dissection product those of the peripheral arm (N‐ and Q modules). The 2D gel of complex I dissection products from Arabidopsis was taken from figure 4 of Klodmann et al. (2010), right image; the intact holo‐complex at 1000 kDa was added using the left image of the same figure.

The determined identities of complex I subunits also enabled interpreting the complex I subcomplexes of 850, 230, 200, and 170 kDa (Figure 6): The 230, 200, and 170 kDa subcomplexes include the subunits of the N module of the peripheral arm (depending on the size of these complex I subcomplexes, the N module is more or less complete). The large 850 kDa subcomplex comprises the subunits of the P_D_, P_P_, and Q modules, including subunits of the carbonic anhydrase module. We conclude that destabilization of *P. tricornutum* complex I causes its dissection into a large 850 kDa subcomplex representing the P_D_, P_P_, and Q modules and three small subcomplexes that represent different versions of the N module (see GelMap at https://www.gelmap.de/phaeo‐ci/). This is in contrast to the situation in *A. thaliana*, where destabilization of complex I leads to two large subcomplexes, one representing the peripheral arm (the N and Q modules) and one the membrane arm (the P_D_ and P_P_ modules) – for comparison see Figure 7.

### Search for further complex I subunits encoded by the *P. tricornutum* genome

**Figure 8:**
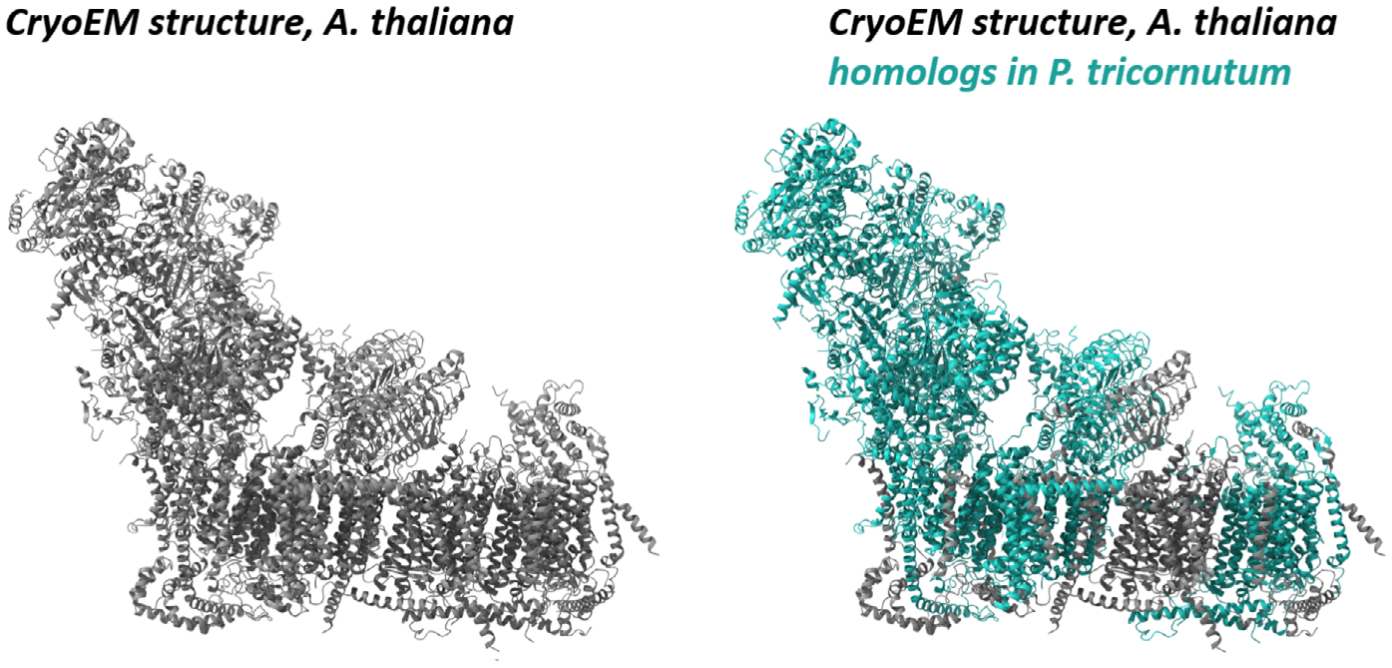
Visualization of the identified *P. tricornutum* complex I subunits within the complex I structure of *Arabidopsis thaliana* [taken from (Klusch et al., 2021); PDB structure 7ARB]. *Left*: CryoEM structure of *Arabidopsis thaliana* complex I (Klusch et al., 2021). *Right*: Homologous *P. tricornutum* complex I subunits identified by MS analyses (organellar and complex I‐enriched fractions) highlighted in the Arabidopsis structure (green). Identities of subunits are given in Figure S7.

*A. thaliana* complex I consists of overall 48 subunits (Klusch et al., 2021, 2023). Using the *P. tricornutum* genome sequence, we systematically searched for homologous subunits. Besides the 30 subunits identified by mass spectrometry in our complex I‐enriched fraction, we identified candidates for another 15 complex I homologs; however, partially with low probability values (Figures S7 and S8). These subunits were not found proteomically, for which there may be various explanations. Some of the corresponding proteins are very small, which means that only a few peptides are generated by trypsinization; other proteins are very hydrophobic, which results in peptides that are difficult to ionize. Further investigations are required to examine if these proteins indeed form part of complex I in *P. tricornutum* (Figure 8).

### Extra complex I subunits in *P. tricornutum*

The proteomic analysis of our complex I‐enriched fraction by 2D BN/SDS PAGE and mass spectrometry also revealed that at least three additional proteins occur in the gel region of complex I, which are not known as complex I subunits in other organisms (named unknown protein 1, unknown protein 2, and unknown protein 3 in our GelMap at https://www.gelmap.de/5850, Figure S6). Their complete sequences could be derived from the *P. tricornutum* protein sequence databases (Figure S8). No functional predictions could be made on the basis of sequence analyses. However, using AlphaFold‐based structural comparisons, unknown protein 2 was identified as a member of the prohibitin family and unknown protein 3 as an ATPase family AAA domain‐containing protein 2B (Table 1). It is known that AAA protease and prohibitin complexes are present in mitochondria. Both have molecular masses of about 1 MDa. We conclude that on our 2D gel, unknown protein 2 and unknown protein 3 likely co‐migrate with complex I, but are not true complex I subunits.

**Table 1:** Identities of the additional *P. tricornutum* proteins identified in the gel region of complex I in the complex I‐enriched fraction (unknown proteins 1, 2, and 3 in Figure 6; Figure S6)

	Protein accession number	Protein length (aa)	*Z*‐score	Predicted protein
1	B7G395	500	17.7	TRANS‐2‐ENOYL‐COA REDUCTASE *H. sapiens*, PDB: 2vcy
			16.3	ALCOHOL DEHYDROGENASE *S. cerevisiae*, PDB: 7kjy
2	B7FWC4	292	16.3	PROHIBITIN *H. sapiens*, PDB: 8rrh
3	B7T8G7	278	2.4	ATPASE FAMILY AAA DOMAIN‐CONTAINING PROTEIN 2B *H. sapiens*, PDB: 3lxj

The three proteins were structurally modeled using AlphaFold. Structures were subsequently used to probe the Protein Database (PDB) using the DALI server (http://ekhidna2.biocenter.helsinki.fi/dali/) (Holm et al., 2023; Holm & Rosenström, 2010). The DALI server reports structural similarities between proteins with *Z*‐scores: the larger the *Z*‐score, the closer the structural match. Values exceeding 2 are generally considered significant and indicate that the proteins are likely to share the same fold. Accession numbers of the putative *P. tricornutum* complex I subunits (given in the column to the left) correspond to those given in the GelMap of the complex I‐enriched fraction at https://www.gelmap.de/phaeo‐ci/. Protein data base (PDB) accessions of the identified structurally similar proteins are given in the column to the right.

**Figure 9:**
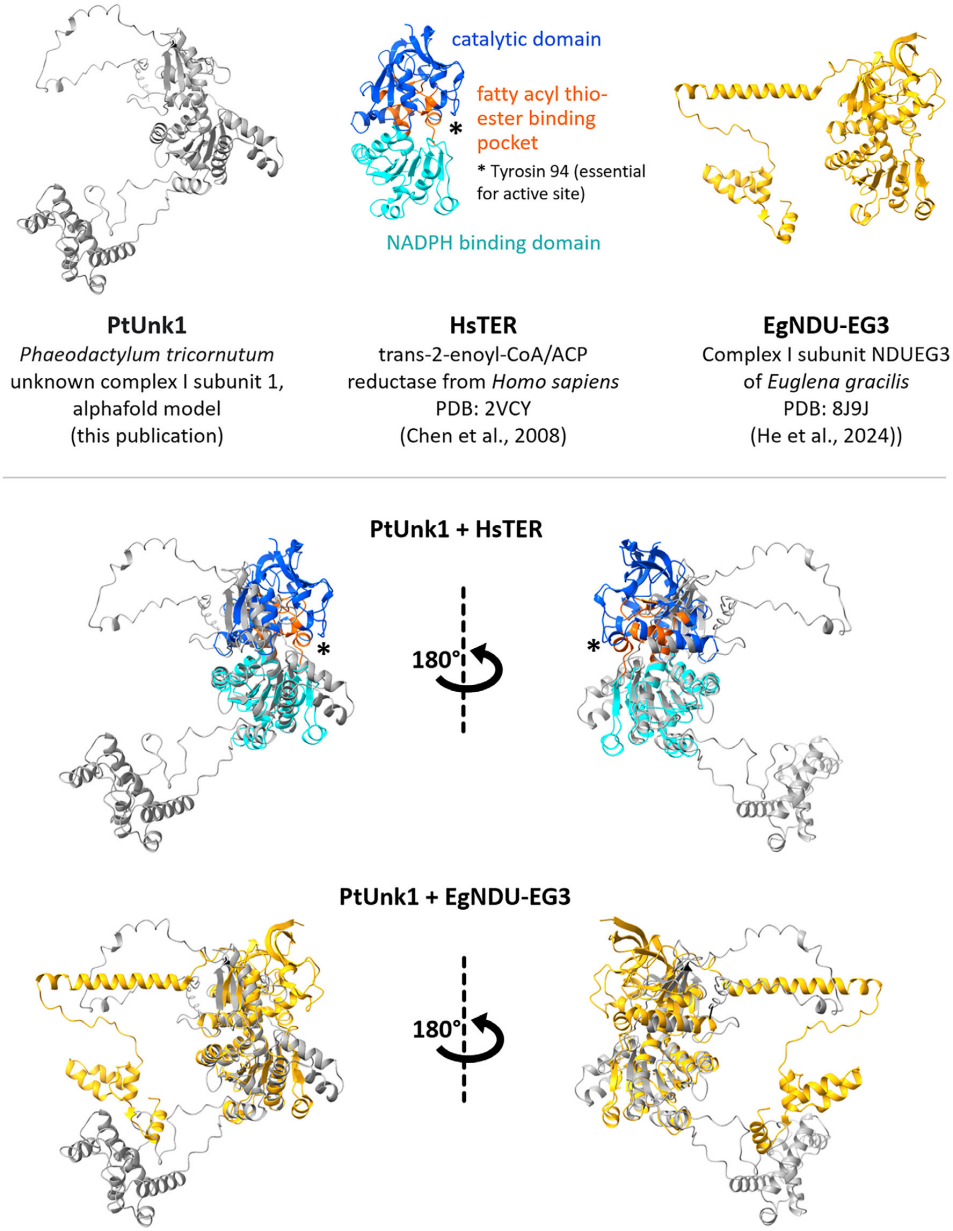
Structural comparison of unknown complex I subunit 1 from *Phaeodactylum tricornutum*, trans‐2‐enoyl‐CoA/ACP reductase from *Homo sapiens*, and *c*omplex I subunit NDUEG3 of *Euglena gracilis*.

In contrast, unknown protein 1 very likely is an extra complex I subunit in *P. tricornutum* (UniProt accession XP_002181748.1/B7G395). It comprises 500 amino acids and was identified with high MS scores, likewise in the 1050 kDa holo‐complex and in the three subcomplexes representing the N module. Comparison of its structure as predicted by AlphaFold with structures available in the Protein Data Base (PDB) using the DALI server (http://ekhidna2.biocenter.helsinki.fi/dali/) revealed that the protein resembles members of a protein superfamily including (i) NAD(P)H‐dependent trans‐2‐enoyl‐CoA/ACP reductases (TER) and (ii) alcohol dehydrogenases (Table 1). Superposition of the predicted structure of unknown protein 1 from *P. tricornutum* and the crystal structure of human NADPH‐dependent mitochondrial enoyl–coenzyme A reductase shows that the N‐terminal Rossmann‐like domain involved in NAD(P)H binding is well conserved, whereas the C‐terminal catalytical domain is less similar (Figure 9). The function of this protein has to be further investigated.

## DISCUSSION

We here report a protocol for the enrichment of complex I from *Phaeodactylum tricornutum*. The starting condition was complicated by the fact that mitochondria form a reticular system on the surface of a large chloroplast in this diatom species. Using proteomics, a total of 1159 proteins could be identified in our organelle‐enriched fraction (Figure 4). A high‐molecular weight complex of 1050 kDa was confirmed as mitochondrial complex I, containing subunits of both the peripheral and the membrane arms, including the carbonic anhydrase module. *P. tricornutum* complex I is slightly larger than its counterpart in animals, fungi, and plants (Kampjut & Sazanov, 2020; Klusch et al., 2021; Parey et al., 2019). Further purification using sucrose gradients yielded a ‘complex I‐enriched’ fraction, which was used to systematically characterize complex I subunits by mass spectrometry. Thirty of the identified subunits are homologous to complex I subunits from other organisms (Figure 6). Candidates for 15 additional conserved complex I subunits in *P. tricornutum* were found by genomic evaluations (Figure S7). Furthermore, we identified at least one subunit that is specific for complex I from *P. tricornutum*. Notably, the disassembly pattern of complex I in this diatom differs from that in Arabidopsis, suggesting differences in the interaction strength between its structural subunits (Figure 7).

In many bacteria, complex I consists of 14 so‐called ‘core’ subunits, which are essential for complex I function. This set of subunits is conserved in the mitochondria of nearly all eukaryotes studied to date. In addition, approximately 25 conserved extra subunits are found in mitochondrial complex I (Senkler et al., 2017). Diatom complex I fits well into this picture. The large number of conserved extra subunits strongly suggests a monophyletic origin of mitochondria, which is supported by many other findings (Gray et al., 1999). However, mitochondrial complex I also contains a few clade‐specific subunits, such as a 42 kDa subunit in mammals that exhibits homology to proteins of the nucleoside kinase family (Vinothkumar et al., 2014) or the sulfur transferase subunit ST1, which forms part of complex I in *Yarrowia lipolytica* (D'Imprima et al., 2016). Also, the complex I‐enriched fraction from *P. tricornutum* includes proteins that were not reported to form part of complex I in other groups of organisms, including a prohibitin and an AAA domain ATPase. In both cases, however, it can be assumed that the proteins belong to protein complexes that co‐migrate with complex I on the 2D gel system used in our study, as they have native molecular masses in the ~1 MDa range (Ahn et al., 2006; Merkwirth & Langer, 2009) and should therefore not be regarded as complex I subunits.

In contrast, another additional protein (UniProt accession XP_002181748.1/B7G395) identified in the complex I‐enriched fraction from *P. tricornutum* fulfills all criteria to be classified as a true complex I subunit (termed unknown subunit 1 in Figure 6; Figures S7 and S8): It is present in the holo‐complex I as well as in the 170, 200, and 230 kDa dissection products of complex I, which all correspond to the N module of the peripheral arm (Figure 6). The complete sequence of the protein can be derived from genomic *P. tricornutum* data and comprises 500 amino acids (Figure S8). Comparisons of its sequence with sequences from public protein databases do not allow reliable predictions of its function. The 3D structure, however, as predicted by AlphaFold2 modeling, resembles the ones of the trans‐2‐enoyl‐CoA/ACP reductase (TER) protein family, which in mitochondria are linked to fatty acid elongation and which use NADPH as electron donor (Chen et al., 2008).

The structural similarity of this diatom complex I subunit to NAD(P)H‐dependent trans‐2‐enoyl‐CoA/ACP reductases family members is intriguing because it has also been reported for complex I from other organisms that subunits of the fatty acid synthase (FAS) are bound to complex I. In *Euglena gracilis*, five subunits (NDUEG1, NDUEG2, NDUEG3, NDUEG5, and NDUEG12) form an integrated FAS module associated with the N module of complex I (He et al., 2024). NDUEG3 and NDUEG5 both exhibit structural similarities with NADPH‐dependent trans‐2‐enoyl‐CoA/ACP reductases from other species. In *Tetrahymena* complex I, a subunit homologous to *E. gracilis* NDUEG2 has been detected (NDUTT2) (Han et al., 2023; Mühleip et al., 2023; Zhou et al., 2022).

Superposition of the predicted *P. tricornutum* unknown protein 1 structure with the crystal structure of human NADPH‐dependent mitochondrial enoyl–coenzyme A reductase reveals conservation of the N‐terminal Rossmann‐like domain [which is involved in binding NAD(P)H] and also the fatty acyl thioester binding pocket, but the catalytic domain differs (Figure 9). Superposition of unknown protein 1 with the *E. gracilis* NDUEG3 subunit also clearly reveals structural similarities, again more in the region of the NAD(P)H binding domain and the substrate binding pocket than in the catalytic domain (Figure 9). In *E. gracilis*, FAS activity of isolated complex I could not be demonstrated (He et al., 2024). The interconnection between mitochondrial complex I and mitochondrial fatty acid biosynthesis has to be further investigated. In this context, it should be noted that mitochondrial complex I of all organisms studied to date contains acyl‐carrier proteins (ACPs) at two positions. Fatty acid biosynthesis at these subunits has been demonstrated for the fungus *Neurospora crassa* (Zensen et al., 1992). Furthermore, in plant and green algae complex I, butyryl or crotonyl‐CoA, both intermediates of fatty acid biosynthesis, have been identified bound to the carbonic anhydrase module (Klusch et al., 2023). Taken together, all these observations indicate that complex I and mitochondrial fatty acid synthase likely were connected during mitochondrial evolution and still are today, at least in some groups of species.

In terms of phylogenetic relationships, diatoms are classified as belonging to the Stramenopiles/Alveolates/Rhizaria (SAR) clade (Shin et al., 2025; Williamson et al., 2025). For this clade, only the complex I structure of *Tetrahymena* has been elucidated to date (Han et al., 2023; Mühleip et al., 2023; Zhou et al., 2022). In this species, complex I is particularly large and characterized by the presence of about 20 additional subunits. However, we were unable to find homologs for these additional subunits in the *P. tricornutum* protein database (results not shown). In fact, our biochemical characterization of complex I from *P. tricornutum* rather revealed that its subunit composition is particularly similar to that of complex I from the Archaeplastida clade (including plants, green algae, red algae, glaucophytes; Figure 8), which supports the view that, in addition to plastids, mitochondria also probably originated from a secondary endosymbiosis event. Indeed, the sequences of the mitochondrial‐encoded subunits from *P. tricornutum* are especially similar to those from red algae (data not shown).

### The carbonic anhydrase module of *P. tricornutum* complex I

According to current knowledge, the carbonic anhydrase module and the bridge module, which comprises an unusual ferredoxin, are absent in mitochondrial complex I from animals and fungi. However, it can be assumed that these modules originally were present in all eukaryotic clades, as they have been described not only for plants and green algae, but also for all protists and amoeba investigated in this respect (Gawryluk et al., 2012; He et al., 2024; Zhou et al., 2022). It has previously been reported that carbonic anhydrase subunits homologous to those found in complex I from plants and protists are also present in diatoms (Cainzos et al., 2021). Furthermore, we here also report the identification of the complex I‐integrated ferredoxin subunit in a diatom.

The carbonic anhydrases occurring in complex I belong to the γ class. In bacteria, γ‐type carbonic anhydrases form homotrimers (Alber & Ferry, 1994). The active centers are formed within the trimer at the three subunit interphases and bind a zinc ion, respectively. In contrast to bacteria, the carbonic anhydrase module of mitochondrial complex I consists of a heterotrimer. In Arabidopsis, there are five genes that code for subunits of the complex I‐integrated carbonic anhydrases. Three encode proteins in which the Zn^2+^‐binding amino acid positions are conserved; they are referred to as γCA1, γCA2, and γCA3 (Parisi et al., 2004). The other two genes code for proteins in which some Zn^2+^‐binding amino acids are missing. These two proteins are called carbonic anhydrase‐like proteins 1 and 2 [γCAL1 and γCAL2 (Perales et al., 2004)]. The CryoEM structure of Arabidopsis complex I revealed that the γCA heterotrimer at complex I consists of two γCA proteins (mainly γCA1 and γCA2) and one γCAL protein [predominantly γCAL2 (Klusch et al., 2021)]. Zn^2+^ can only bind at the γCA1‐γCA2 interphase.

The original names of γCAs in diatoms were introduced by Samukawa et al. (2014) for *Thalassiosira pseudonana*, but homologous proteins are found in *P. tricornutum*. However, we here suggest to re‐name the *P. tricornutum* γCA/γCAL proteins in accordance with their similarity to the homologous Arabidopsis proteins because these γCA/γCAL subunits were the ones discovered first within complex I (Parisi et al., 2004; Perales et al., 2004) and because they are the most extensively characterized complex I‐integrated γCA/γCAL proteins (see Fromm et al., 2016 for review on the γCA domain; see Figure S7 for subunit naming; Figure S9 for an alignment of the five γCA/γCAL proteins from *P. tricornutum*; Figure S10 for a sequence similarity tree of γCA/γCAL proteins from *A. thaliana* and *P. tricornutum*).

The proteomic analysis of our complex I‐enriched fraction from *P. tricornutum* revealed identification of one γCA and one γCAL subunit. The sequences of the corresponding genes are known. Two different genes were identified for Pt γCAL (UniProt accessions B7GE24 and B7GE20), which encode almost identical proteins. Both proteins, previously named ‘CA3’ in diatoms, exhibit the highest sequence similarity with the Arabidopsis γCAL1 protein (however, the similarity of this protein to the Arabidopsis CAL2 protein is almost the same). We term these proteins Pt γCAL‐1 and Pt γCAL‐2 (Figure S9). Similarly, for the *P. tricornutum* γCA protein, previously named ‘CA4’ in diatoms, there are two database entries (Figure S9). Based on structural similarities to the γCA2 proteins from other organisms we term these proteins Pt γCA2‐1 and Pt γCA2‐2. This naming also is based on the fact that the protein occupies the same position within the γ‐carbonic anhydrase trimer as the γCA2 protein from plants (Cainzos et al., 2021; although still referred to as γCA4 in this publication) and that it is particularly abundant in comparison to other γCA subunits of complex I. Pt γCA2‐2, encoded by UniProt entry B7FY50, consists of 175 amino acids and has a calculated molecular weight of 18.03 kDa. It is substantially smaller than γCA homologs in plants (which are approximately 30 kDa) and in other diatoms, such as *Halamphora coffeaeformis*, which also includes a gene encoding a γCA homolog of around 30 kDa (Cainzos et al., 2021). The DiatOmicBase sequence database (www.diatomicsbase.bio.ens.psl.eu) includes another *P. tricornutum* γCA2 entry (gene accession: Phatr3_EG02042), which encodes a protein of 278 amino acids with a predicted molecular weight of 29.19 kDa. This protein, termed Pt γCA2‐1 by us (Figure S9), is almost identical to Pt γCA2‐2 (encoded by B7FY50), but carries extensions at its N‐ and C‐termini. Since the protein identified as Pt γCA2 on our 2D gels migrates at ~30 kDa (Figure S6), we conclude that the entry B7FY50 (encoding Pt γCA2‐2) likely only represents part of the Pt γCA2 protein, and that Pt γCA2‐1 represents the complete Pt γCA2 protein.

Another gene encoding a γCA protein in *P. tricornutum* (accession B5Y401) was identified genomically, which we term Pt γCA1 and which shows less similarity to Arabidopsis γCAs (Figures S9 and S10). However, this protein could not be identified proteomically in our complex I‐enriched fraction. We conclude that the heterotrimeric γCA module of *P. tricornutum* possibly consists of two copies of the γCA2 protein and one copy of a γCAL protein. However, structural modeling of a heterotrimeric γCA domain of γCA2/γCA2/γCAL1 composition reveals that none of the three putative active sites has a complete set of Zn^2+^‐binding residues (data not shown). We currently cannot rule out the possibility that the γCA1 protein is also synthesized in *P. tricornutum* and integrated into complex I, but has escaped proteomic identification. A γCA heterotrimer with γCA1/γCA2/γCAL1 composition could bind Zn^2+^ at the γCA1/γCA2 interphase consistent with what has been proposed in another pennate diatom species, *Halamphora coffeaeformis* (Cainzos et al., 2021). The clarification of this issue will require further structural analysis.

The function of the γCA module of complex I is still unclear. It has been suggested that the protons released during the formation of bicarbonate from water and CO_2_ are available to the membrane arm of complex I for proton translocation across the inner mitochondrial membrane (Braun & Klusch, 2024). In this context, a promoting effect of the ferredoxin subunit, which is located within the bridge module that connects the CA module and the peripheral complex I arm, is discussed. In our study, this complex I‐integrated ferredoxin was identified for the first time in a diatom, which provides new evidence that the bridge module of complex I has to be regarded as an original feature of eukaryotic complex I.

### Outlook

This study provides a foundation for understanding the mitochondrial electron transport chain in diatoms, with a particular focus on complex I, the largest and most intricate of the five OXPHOS complexes. The purification protocol presented here enables efficient enrichment of *P. tricornutum* complex I and offers the prospect of its structural characterization, for example, by cryogenic single particle electron microscopy. Future structural analysis will advance our understanding of respiratory chain evolution in photosynthetic protists compared to plants, algae, and non‐photosynthetic organisms, and may uncover new targets for metabolic engineering.

## MATERIALS AND METHODS

### Cultivation of *P. tricornutum*

*Phaeodactylum tricornutum* strain SAG 1090–6 obtained from SAG (Culture Collection of Algae at Göttingen University, Germany, https://uni‐goettingen.de/en/45175.html) was cultivated in liquid f/2 medium (Guillard, 1975) supplemented with 40 mm NO_3_. Cultures were grown under illumination of 80 μmol m^−2^ sec^−1^ (True Daylight white‐LED) at 21°C and constant light. Pre‐culture volumes up to 50 ml were constantly agitated at 150 rpm. Liquid culture of up to 2 L was aerated with sterile compressed air, which also promoted its mixing.

### Preparation of organelle‐enriched fractions

*P. tricornutum* cells (strain SAG 1090‐6), harvested from a 2 L culture with an optical density (OD_600_) of approximately 20, were used as starting material for the preparation of organelle‐enriched fractions. Cells were spun down in a Sorvall Lynx 6000 centrifuge using a F12 Fiberlite rotor (Thermo Scientific, Bremen, Germany) at 5000 ***g*** and 4°C for 5 min, with a deceleration of 7. After this centrifugation step, the pellet was resuspended in f/2 medium and transferred to 50 ml‐falcon tubes. Samples were again centrifuged, applying the same parameters (BioFlex HC rotor; Thermo Scientific). The resulting pellet was resuspended in the smallest possible volume of cell disruption buffer (0.3 m sucrose, 60 mm TES, 25 mm sodium pyrophosphate, 4 mm EDTA, 10 mm KH_2_PO_4_, 1 mm glycine, 1% [w/v] polyvinylpyrrolidone 40, 1% [w/v] BSA, 50 mm sodium ascorbate, 20 mm cysteine, pH 8.0 [KOH]). For all following steps, the fractions were kept at 4°C/on ice. *P. tricornutum* cells were disrupted twice by French press treatment (SLM Aminco FA‐078 French pressure cell press; SLM Instruments, Inc., Urbana, IL, USA), applying maximum pressure (130 MPa). After repeating this step, cell disruption was continued by mortar treatment with sea sand for 6 min. The enrichment of *P. tricornutum* organelles followed a method for enriching mitochondria from Arabidopsis leaves (Keech et al., 2005), with modifications. Disrupted cells were centrifuged twice at 2500 ***g*** (Sorvall Lynx 6000, F14 Fiberlite rotor; Thermo Scientific) for 5 min (to remove cell debris and sand) and afterward centrifuged once at 15 100 ***g*** for 15 min to sediment organelles. The pellet was resuspended in 12 ml washing buffer (0.3 m sucrose, 10 mm TES, 10 mm KH_2_PO_4_, pH 7.5 [KOH]), homogenized by two gentle strokes in a 15 ml Dounce homogenizer, and finally transferred onto twelve 19 mL‐Percoll gradients (0.3 m sucrose, 10 mm TES, 1 mm EDTA, 10 mm KH_2_PO_4_, 1 mm glycine, 50% [v/v] Percoll, pH 7.5 [KOH]). Centrifugation was carried out at 17 400 ***g*** (Sorvall WX Ultra, T‐1250 rotor; Thermo Scientific) for 40 min, applying minimum acceleration and deceleration speeds. The gradients were fractionated according to visible bands. Washing steps of recovered fractions (for Percoll removal) were performed in washing buffer at 18 000 ***g*** for 20 min (Sorvall Lynx 6000, A27 rotor; Thermo Scientific). The pellets obtained for each fraction were resuspended in washing buffer (1 g pellet per milliliter), and then transferred into Eppendorf tubes (1 ml suspension each). The samples were either used directly or frozen in liquid nitrogen and stored at −80°C.

### 2D Blue native (BN)/SDS PAGE

The Protean II protein gel electrophoresis system (Biorad, Feldkirchen, Germany) was used for protein separations by electrophoresis. 2D Blue native (BN)/SDS polyacrylamide gel electrophoresis (PAGE) was carried out as described previously (Wittig et al., 2006). In short, for the first gel dimension, polyacrylamide gradient gels were casted from bottom to top at 4°C using a gradient mixer by combining a low‐concentration acrylamide solution (4.5% [v/v] acrylamide in gel buffer BN [250 mm amino caproic acid, 25 mm Bis‐Tris, pH 7.0]), and a high‐concentration acrylamide solution (16% [v/v] acrylamide, 19% [v/v] glycerol in gel buffer BN). After polymerization, sample gels (4% acrylamide in gel buffer BN) were poured on top of the gradient gels and gel pockets were generated by using a gel‐comp. Organelle fractions of interest (about 500 μg protein) were pretreated with digitonin (5 mg/mg protein) for membrane protein solubilization, supplemented with a Coomassie blue solution (5% [w/v] Coomassie blue in 750 mm amino caproic acid; final Coomassie concentration ~0.25% [w/v]) and loaded into the pockets of the gels. Anode and cathode buffers were added to the gel chambers and BN PAGE was conducted at 4°C in a two‐step setup (first step: 100 V, 15 mA, 45 min; second step: 15 mA, 500 V, 13 h). BN gels were either stained with Coomassie colloidal (see below) or used for SDS PAGE as a second gel dimension according to Wittig et al. (2006).

For second gel dimension, gel lanes were excised from the BN gels and incubated for 30 min in a 1% SDS / 1% beta‐mercaptoethanol solution. After removal of this solution with water, a lane of a BN gel was placed on a glass plate at the position of the teeth of a gel comb. Gel spacers and the second glass plate were added and everything assembled into the gel‐casting stand. A three‐phase acrylamide gel was poured into the space between the two glass plates: first the *separation gel* (16% [v/v] acrylamide, 12% [v/v] glycerol in gel buffer SDS [1 m Tris, 0.1% SDS, pH 8.45]); afterwards the smaller *spacer gel* (10% [v/v] acrylamide in gel buffer SDS) and finally, after polymerization of the first two gel phases, the *sample gel* (10% [v/v] acrylamide, 10% [v/v] glycerol, 0.1% [w/v] SDS in gel buffer BN), which embeds the BN gel lane and connects it to the other gel phases. After adding anode buffer (0.2 m Tris, pH 8.9) and cathode buffer (0.1 m Tris, 0.1 m tricine, 0.1% SDS pH 8.45) to the gel chamber, electrophoresis was carried out for approximately 18 h at 30 mA. Resulting 2D gels were stained with Coomassie colloidal.

### Colloidal Coomassie staining

Proteins were visualized on gels either by the standard Coomassie Brilliant Blue colloidal staining method (Neuhoff et al., 1988) or by Blue Silver (Candiano et al., 2004).

### In gel complex I activity

After separation of protein complexes by BN PAGE, single gel lanes were excised and washed for 10 min with distilled water. NADH‐dehydrogenase activity of complex I was verified by incubating the gel lanes in an activity solution containing 0.1 m Tris–HCl (pH 7.4), 0.14 mm NADH, and 0.5 mg ml^−1^ nitro blue tetrazolium (NBT). The incubation was carried out for 5 to 30 min, until purple‐colored bands appeared, indicating enzymatic activity. The reaction was stopped by adding fixing solution containing 15% (v/v) ethanol and 10% (v/v) acetic acid. As a control, mitochondria isolated from *Arabidopsis thaliana* wild type cell culture were used. Arabidopsis mitochondria were purified by differential centrifugation and Percoll density step gradient centrifugation as described before (Werhahn et al., 2001).

### Sucrose gradient ultracentrifugation for complex I enrichment

Organelle‐enriched fractions (1 ml) (prepared as described above) were pelleted by centrifugation at 14 300 ***g*** and 4°C for 10 min and subsequently resuspended in 1 ml of digitonin solubilization buffer (30 mm HEPES pH 7.4, 150 mm potassium acetate, and 5% [w/v] digitonin). Incubation took place for 15 min on ice. After centrifugation at 18 300 ***g*** for 10 min at 4°C, solubilized protein complexes were transferred onto 15 ml‐sucrose gradients (0.3–1.5 m sucrose in gradient buffer, 30 mm HEPES [pH 7.8], 150 mm potassium acetate, and 0.1% [w/v] digitonin) and separated by ultracentrifugation at 146 000 ***g*** and 4°C for 20 h. Subsequently, sucrose gradients were fractionated and protein contents of relevant fractions were monitored by 1D BN PAGE and 2D BN/SDS PAGE.

### Protein identification by mass spectrometry and protein data processing

#### *In‐gel* digestion with trypsin

*In‐gel* digestion with trypsin was carried out as described before (Klodmann et al., 2010). Coomassie‐stained protein spots were cut from 2D BN/SDS gels, washed with ultrapure water, and dehydrated by incubation in 100% acetonitrile (ACN). ACN treatment was repeated after each of the following incubation steps to fully remove each solution. Spots were treated first with dithiothreitol (20 mm in 0.1 m NH_4_HCO_3_) for 30 min at 56°C to reduce disulfide bridges and afterwards with iodoacetamide (55 mm in 0.1 m NH_4_HCO_3_) for 30 min in the dark to block cysteine residues. After washing the spots in 0.1 M NH_4_HCO_3_ for 15 min at room temperature, they were incubated with sequencing grade modified trypsin (0.4 μg ml^−1^ [Promega] in 0.1 m NH_4_HCO_3_) at 37°C overnight. Resulting peptides were extracted by washing the gel pieces in a 1:1 mixture of ultrapure H_2_O and acetonitrile with 5% formic acid (FA) (once) and 0.1% FA (twice). In the last step, 100% acetonitrile was used to extract all remaining solution. Peptides were vacuum‐dried and used for mass spectrometric analyses (see below).

Two different mass spectrometry setups were used. For the 2D gels of organelle‐enriched fractions, analyses were performed using an U3000 HPLC – Q Exactive MS (Thermo Scientific) setup. For the 2D gels of the complex I‐enriched fractions, due to lower sample amounts, we applied a more sensitive approach using an Evosep One HPLC (Evosep, Odense, Denmark) coupled to a TimsTOF Pro mass spectrometer (Bruker, Bremen, Germany).

#### Mass spectrometry analysis and data evaluation of proteins of the ‘organelle‐enriched fraction’

Peptides obtained after *in‐gel* digestion and extraction were resuspended in 20 μl solution P (5% [v/v] ACN, 0.1% [v/v] trifluoroacetic acid), incubated for 10 min in an ultrasonic bath, centrifuged for 10 min at 21 000 ***g*** and carefully transferred to glass vials for storage in an auto sampler at 4°C.

HPLC‐ESI‐Q‐Orbitrap measurements were performed with an online U3000 HPLC – Q Exactive MS (Thermo Scientific) setup and coordinated using the Xcalibur software (Thermo Scientific). For HPLC separation, a two‐column setup was used with a 2 cm pre‐column (Acclaim PepMap™ 100, 75 μm × 2 cm, C18, 3 μm, 100 Å; Thermo Scientific) combined with a 50 cm analytical column (Acclaim PepMap™ 100, 75 μm × 50 cm, C18, 3 μm, 100 Å; Thermo Scientific). Column oven temperature was set to 45°C. After sample loading (5 μl) onto the pre‐column, a gradient suitable for peptides from 2D spots was applied. The gradient started with 5% (v/v) ACN in 0.1% (v/v) FA for 5 min, then gradually increased to 40% ACN (v/v) in 0.1% (v/v) FA after 20 min runtime and further increased to 76% ACN (v/v) in 0.1% (v/v) FA at 23 min runtime. From minute 23 to 25, 76% ACN (v/v) in 0.1% (v/v) FA was kept and then strongly reduced to the starting concentration of 5% at minute 26. To equilibrate the column, this concentration was kept for another 10 min. Eluting peptides were ionized using a NSI source (Thermo Scientific) equipped with a stainless‐steel nano‐bore emitter at a capillary voltage of 2.2 kV and a temperature of 275°C. Radio frequency level of the S‐lens was set to 50%. The MS was operated in a positive ion mode and analyses were performed using a top 10 data‐dependent acquisition (DDA) strategy. Only ions with a charge between 2 and 5 were considered. The m/z‐range for full MS scans was 400–1600 m/z at a resolution of 70 000. The AGC target was set to 1e^6^ at a maximum injection time of 120 ms. Top 10 peptides were fragmented using a normalized collision energy of 27%. MS/MS scans were recorded at a resolution of 17 500 and an isolation width of 3 m/z, using an AGC target of 1e^5^ and a maximum injection time of 120 ms. Dynamic exclusion was set to 60 sec.

Acquired spectra were queried against a *Phaeodactylum tricornutum* database obtained from UniProt (www.uniprot.org, Proteome ID UP000000759, downloaded on August 15th, 2025), containing canonical and isoform sequences, using the MaxQuant software version 2.6.8.0 (Cox & Mann, 2008). Default parameters were applied and additionally, the ‘iBAQ’ function was enabled, allowing computation of intensity based absolute quantification (iBAQ) values to estimate abundance of identified proteins (Schwanhäusser et al., 2011). Before searching, sequences of *Phaeodactylum tricornutum* carbonic anhydrases and carbonic anhydrase‐like proteins forming subunits of mitochondrial complex I were added to the database. Resulting protein and peptide data were transferred to Excel (Microsoft Office) for further evaluation.

#### Generation of a GelMap for the ‘organelle‐enriched fraction’

Identified proteins were functionally categorized based on sequence comparisons. Digital GelMaps were generated following the instructions at the GelMap website at www.gelmap.de/create. The GelMap for the organelle‐enriched fraction of *Phaeodactylum tricornutum* is available at https://www.gelmap.de/phaeo‐organelles/.

#### Mass spectrometry analysis and data evaluation of proteins of the ‘complex I‐enriched fraction’

Peptides obtained after *in‐gel* digestion and extraction were resuspended in 20 μl solution A (0.1% [v/v] FA) and prepared for Evosep HPLC separation using Evotips (Evosep) according to the manufacturer instructions.

Peptides were separated by liquid chromatography on an Evosep One (Evosep) equipped with a 4 cm reverse phase C18 column (inner diameter 150 μm, bead size 1.9 μm [EV1107, Evosep]) using the 500 samples per day (SPD) method. Eluting peptides were transferred to a TimsTOF Pro mass spectrometer (Bruker, Bremen, Germany) using a captive spray ion (CSI) source with a capillary voltage of 1600 V and a dry temperature of 180°C. The instrument was operated in data‐dependent acquisition (DDA), parallel accumulation serial fragmentation (PASEF) mode using the pre‐installed short gradient method with a cycle time of 0.5 sec, scan range of 100–1700 m/z and a mobility window from 0.85 to 1.3 V sec^−1^ cm^−2^. Only ions with a charge between 1 and 5 were considered, with an active exclusion of 0.4 min. Fragmentation energy for MS/MS scans was set to 20 eV for peptides with a collisional cross section of 0.6 V sec^−1^ cm^−2^ and to 59 eV for a collisional cross section of 1.6 V sec^−1^ cm^−2^. Collision energies were linearly interpolated between these values.

Data evaluation was carried out with MaxQuant (version 2.6.8.0), as outlined above for the ‘organelle‐enriched fraction’.

#### Generation of a GelMap for the ‘complex I‐enriched fraction’

Identified proteins were compared to complex I subunits of other species and named according to the nomenclature used for Arabidopsis (Klusch et al., 2021, 2023). A digital GelMap was generated following the instructions at the GelMap website at https://www.gelmap.de/create. The GelMap for the complex I‐enriched fraction of *P. tricornutum* is available at https://www.gelmap.de/phaeo‐ci/.

#### Protein structure modeling and structure‐based protein comparison

AlphaFold (https://alphafoldserver.com) was used to model the structure of the three unknown proteins present in our *P. tricornutum* complex I‐enriched fraction. Furthermore, the obtained structures were used for structural comparisons with entries in the Protein Data bank (PDB, https://www.rcsb.org/) (Berman et al., 2000) using the DALI server (http://ekhidna2.biocenter.helsinki.fi/dali/) (Holm et al., 2023; Holm & Rosenström, 2010).

## CONFLICT OF INTEREST

The authors declare no conflict of interest.

## Supporting information


**Figure S1.** Comparison of six different experimental strategies for organelle isolations from *P. tricornutum*.
**Figure S2.** Workflow for isolation of an organelle‐enriched fraction from *P. tricornutum*.
**Figure S3.** Evaluation of the protein composition of the organelle‐enriched fraction by cumulated protein quantities (iBAQ values) assigned to subcellular compartments according to SUBAcon (https://suba.live/).
**Figure S4.** Workflow for isolation of complex I from *P. tricornutum*.
**Figure S5.** Enrichment of *P. tricornutum* complex I by sucrose gradient ultracentrifugation.
**Figure S6.** GelMap of complex I from *Phaeodactylum tricornutum*.
**Figure S7.** Complex I subunits of *A. thaliana* and their (putative) homologs in *P. tricornutum*.
**Figure S8.** Additional proteins identified in the gel region of complex I (https://www.gelmap.de/phaeo‐ci/), which do not resemble known complex I subunits of other organisms.
**Figure S9.** Multiple sequence alignment of *P. tricornutum* γ‐type carbonic anhydrases and their coverage by detected peptides.
**Figure S10.** Sequence similarity tree of the amino acid sequences of the γCA and γCAL subunits of mitochondrial complex I encoded by the genomes of *A. thaliana* and *P. tricornutum*.
**Table S1.** MS data of the GelMap of the organelle‐enriched fraction of *Phaeodactylum tricornutum*.
**Table S2.** Complex I subunits of *Phaeodactylum tricornutum* (extended list).

## Data Availability

*MS data*: All LC–MS/MS raw files acquired in this study, as well as the corresponding MaxQuant search engine results, can be downloaded from the MassIVE repository (University of California San Diego, CA, USA, http://massive.ucsd.edu). *Dataset for the organelle‐enriched fraction*: ftp://massive‐ftp.ucsd.edu/v11/MSV000099308/. *Dataset for the complex I‐enriched fraction*: ftp://massive‐ftp.ucsd.edu/v11/MSV000099310/. *Reference maps*: The GelMap of our organelle‐enriched fraction can be inspected at our GelMap portal at https://www.gelmap.de/phaeo‐organelles/. The GelMap of our complex I‐enriched fraction can be inspected at our GelMap portal at https://www.gelmap.de/phaeo‐ci/.
