# Supplementary material for: From isolation to insights: mitochondrial complex I in the diatom Phaeodactylum tricornutum
Source: Plant J. 2026 Jan 29;125(3):e70706. doi: 10.1111/tpj.70706 (PMC12854811; doi:10.1111/tpj.70706)
Supplement: Supplementary file 1 — Figure S1. Comparison of six different experimental strategies for organelle isolations from P. tricornutum. Figure S2. Workflow for isolation of an organelle‐enriched fraction from P. tricornutum. Figure S3. Evaluation of the protein composition of the organelle‐enriched fraction by cumulated protein quantities (iBAQ values) assigned to subcellular compartments according to SUBAcon (https://suba.live/). Figure S4. Workflow for isolation of complex I from P. tricornutum. Figure S5. Enrichment of P. tricornutum complex I by sucrose gradient ultracentrifugation. Figure S6. GelMap of complex I from Phaeodactylum tricornutum. Figure S7. Complex I subunits of A. thaliana and their (putative) homologs in P. tricornutum. Figure S8. Additional proteins identified in the gel region of complex I (https://www.gelmap.de/phaeo‐ci/), which do not resemble known complex I subunits of other organisms. Figure S9. Multiple sequence alignment of P. tricornutum γ‐type carbonic anhydrases and their coverage by detected peptides. Figure S10. Sequence similarity tree of the amino acid sequences of the γCA and γCAL subunits of mitochondrial complex I encoded by the genomes of A. thaliana and P. tricornutum. Table S1. MS data of the GelMap of the organelle‐enriched fraction of Phaeodactylum tricornutum. Table S2. Complex I subunits of Phaeodactylum tricornutum (extended list). [file TPJ-125-0-s001.zip › tpj70706-sup-0001-FigureS1-S10.docx]

Berdun et al.

Supplementary Material

Supplementary Figure 1: Comparison of six different experimental strategies for organelle isolations from *P. tricornutum*.

Supplementary Figure 2: Workflow for isolation of an organelle fraction from *P. tricornutum*.

Supplementary Figure 3: Evaluation of the protein composition of the organelle-enriched fraction by cumulated protein quantities (iBAQ values) assigned to subcellular compartments according to SUBAcon (https://suba.live/).

Supplementary Figure 4: Workflow for isolation of complex I from *P. tricornutum*.

Supplementary Figure 5: Enrichment of *P. tricornutum* complex I by sucrose gradient ultra-centrifugation.

Supplementary Figure 6: GelMap of Complex I from *Phaeodactylum tricornutum*.

Supplementary Figure 7: Complex I subunits of *A. thaliana* and their (putative) homologs in *P. tricornutum*.

Supplementary Figure 8: Additional proteins identified in the gel region of complex I (<https://www.gelmap.de/phaeo-ci/>), which do not resemble known complex I subunits of other organisms.

Supplementary Figure 9: Multiple sequence alignment of *P. tricornutum* γ-type carbonic anhydrases and their coverage by detected peptides.

Supplementary Figure 10: Sequence similarity tree of the amino acid sequences of the γCA and γCAL subunits from mitochondrial complex I encoded by the genomes of *A. thaliana* and *P. tricornutum*.

Supp. Figure 1


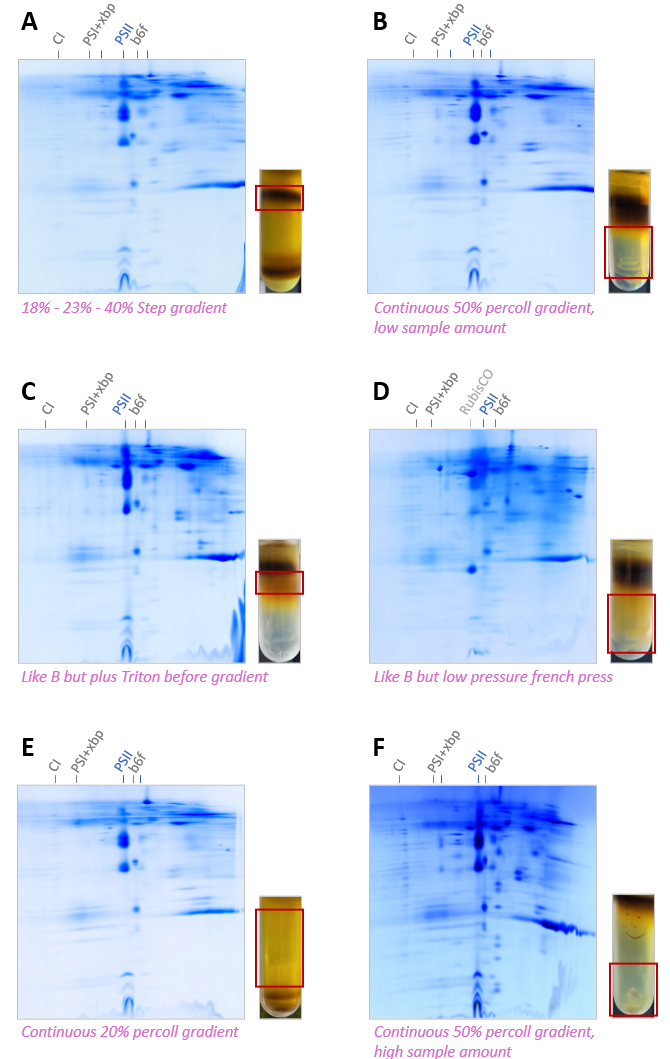


**Supplementary Figure 1*:* Comparison of six different experimental strategies for organelle isolations from *P. tricornutum*.** For each experimental approach, fractions obtained by percoll gradient ultracentrifugation were analyzed by 2D BN / SDS PAGE (only the 2D gel of the best fraction is shown, respectively). Images of the gradients are shown to the right of the 2D gels; the areas of the gradients from which the respective fraction was taken is marked by a red box, respectively. Identities of the protein complexes are indicated on top of the gels (CI: complex I; PSI+xbp: photosystem I + fucoxanthin-binding proteins; PSII: photosystem II; b6f: cytochrome b_6_f complex; RubisCO: ribulose bisphosphate carboxylase/oxygenase). The most abundant protein complex, PSII, is highlighted in blue. Methods shown are: A) step gradient; B) continuous gradient (pre-centrifugated, 50% percoll); C) continuous gradient, pre-treated with 0.1 % Triton; D) continuous gradient after cell disruption at low pressure (20 MPa) during french press treatment; E) continuous gradient (no pre-centrifugation, 20% percoll, as described by (Huang *et al.*, 2024)); F) continuous gradient (no pre-centrifugation, 50% percoll).

Supp. Figure 2


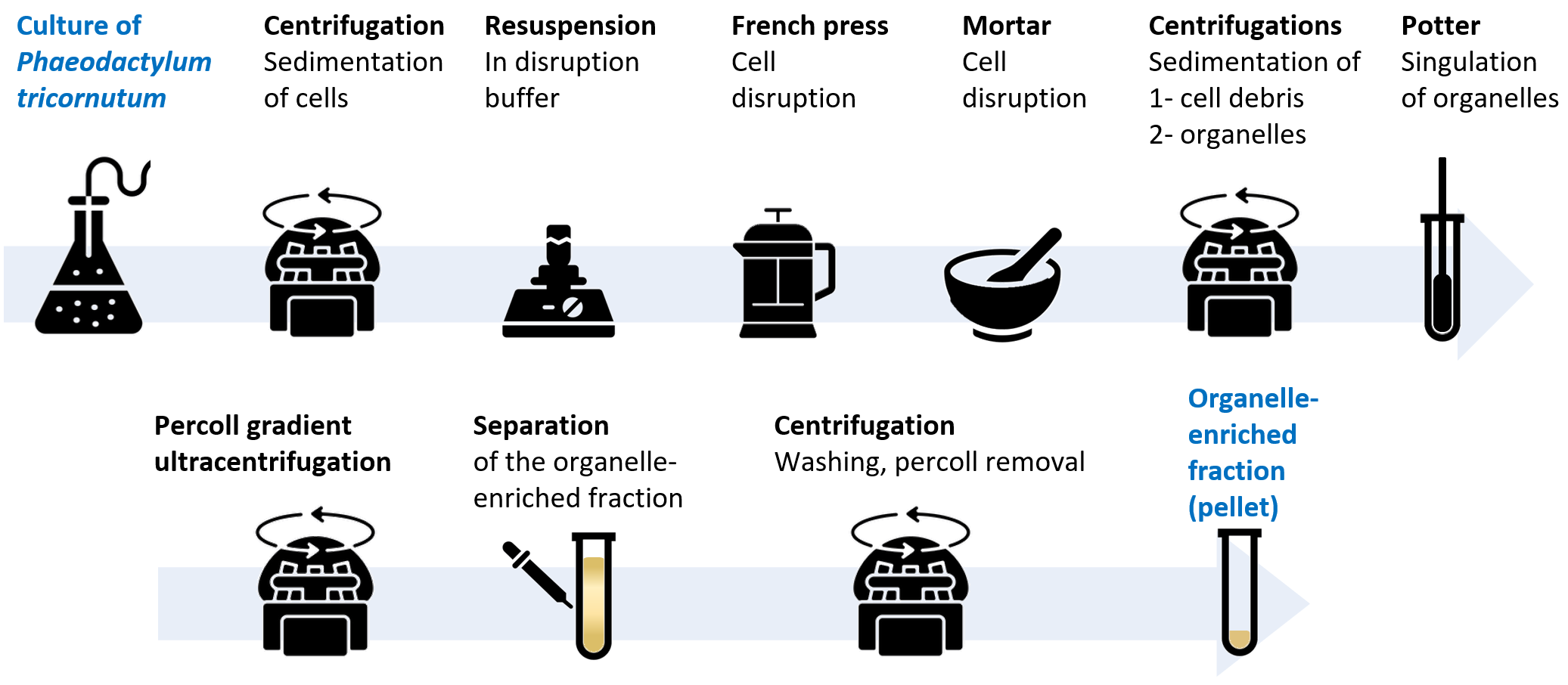


**Supplementary Figure 2: Workflow for isolation of an organelle-enriched fraction from *P. tricornutum*.** *P. tricornutum* cells were harvested by centrifugation. They were resuspended in disruption buffer using a vortexer and disrupted by French press treatment as well as pestling. After cell debris removal by centrifugation, organelles were sedimented, resuspended in washing buffer and dispersed by potter treatment. The resulting homogenate was separated by Percoll density gradient ultracentrifugation. After several washing steps for Percoll removal, an organelle-enriched pellet was obtained. See method section for further details. Several icons for the workflow were taken from the noun project (<https://thenounproject.com>).

Supp. Figure 3


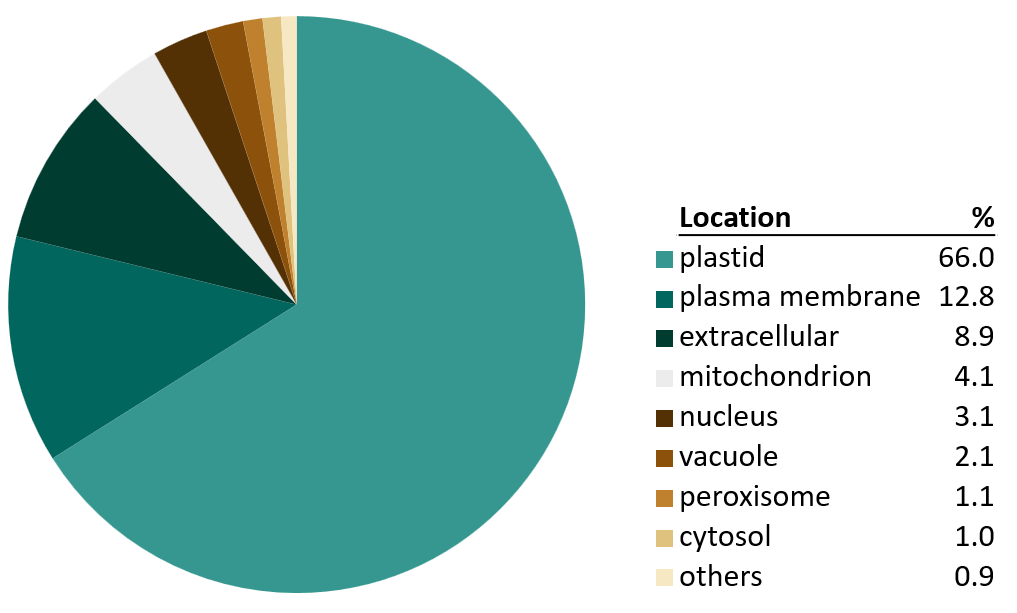


**Supplementary Figure 3: Evaluation of the protein composition of the organelle-enriched fraction by cumulated protein quantities (iBAQ values) assigned to subcellular compartments according to SUBAcon (https://suba.live/).** Proteins of an organelle-enriched fraction of *P. tricornutum* were separated by 2D Blue native / SDS PAGE, visualized by Coomassie-staining and identified by MS (Fig. 3). MS data were searched against a *P. tricornutum* protein database ([www.uniprot.org](http://www.uniprot.org), Proteome ID UP000000759). Overall, 1159 proteins were identified in 95 spots. The pie chart shows the subcellular localization of the identified proteins based on assignment of the homologous Arabidopsis proteins to subcellular compartments using the “Subcellular localization database for Arabidopsis proteins” (SUBA5) at [www.suba.live](http://www.suba.live/). The pie chart was built using sums of iBAQ values per subcellular compartment.

Supp. Figure 4


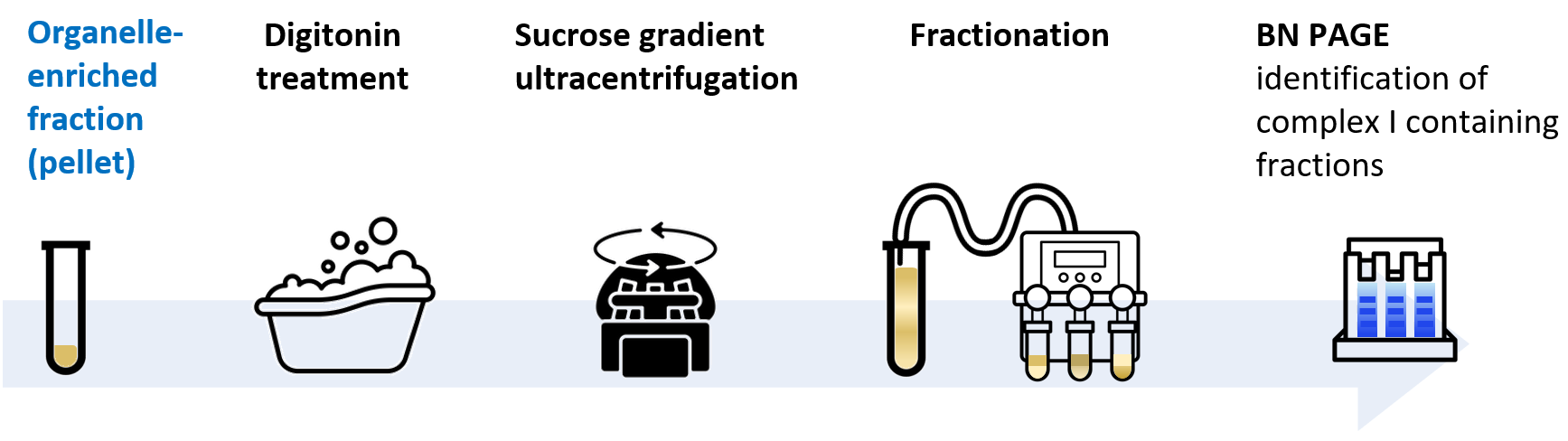


**Supplementary Figure 4: Workflow for isolation of complex I from *P. tricornutum.*** An organelle-enriched fraction (**Supp. Figure 1**) was treated with digitonin for membrane solubilization and separated by sucrose gradient ultracentrifugation (Supp. Fig. 5). After fractionation of the gradient, aliquots of each fraction were analyzed by Blue native (BN) PAGE to identify complex I containing fractions. See method section for further details. Several icons for the workflow were taken from the noun project (<https://thenounproject.com>).

Supp. Figure 5

**
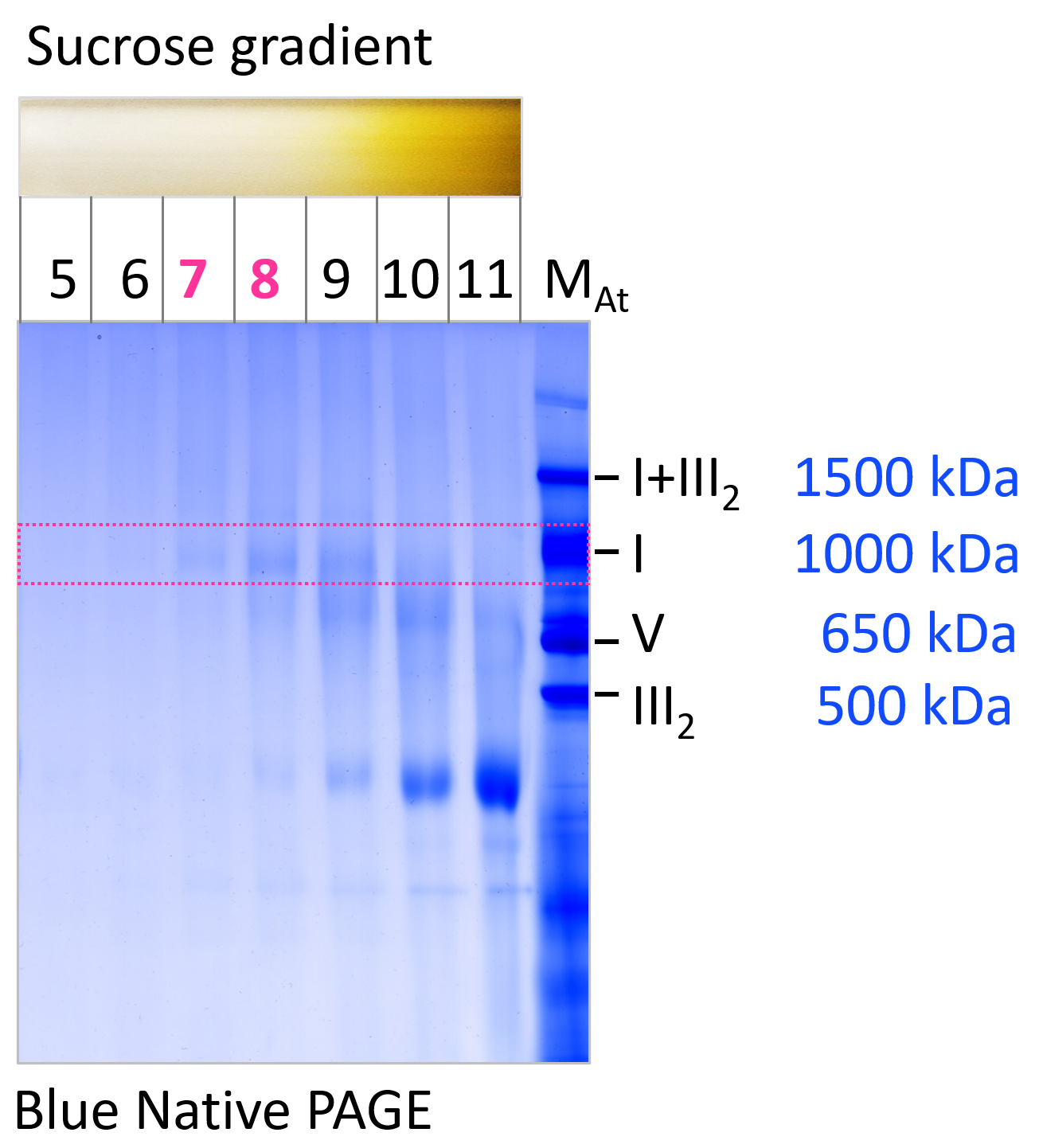
**

**Supplementary Figure 5:** **Enrichment of *P. tricornutum* complex I by sucrose gradient ultracentrifugation.** An organelle-enriched fraction was loaded onto a sucrose gradient and centrifuged at 146,000xg and 4°C for 20 h. The resulting gradient (top) was fractionated from bottom (right) to top (left) and aliquots of relevant fractions were analyzed by 1D Blue native PAGE (bottom) to monitor the protein complex content. A mitochondrial fraction of *Arabidopsis thaliana* was loaded onto the gel for reference (M_At_). On the 1D gel, fractions 7 and 8 are most enriched in complex I. I+III_2_: supercomplex formed of monomeric complex I and dimeric complex III; I: complex I; V: ATP synthase (complex V); III_2_: dimeric complex III.

Supp. Figure 6


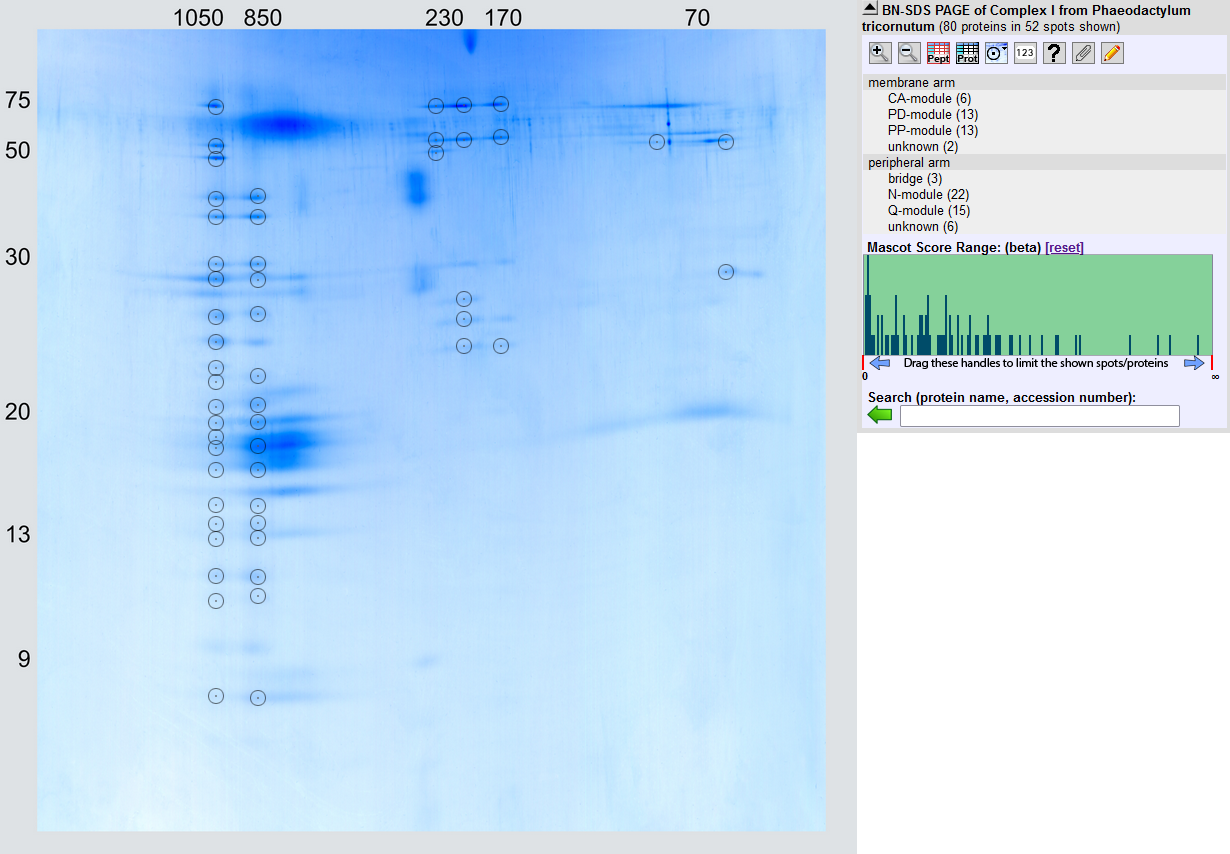


**Supplementary Figure 6: GelMap of Complex I from *Phaeodactylum tricornutum*.** The proteome of a complex I-enriched fraction of *Phaeodactylum tricornutum* was separated by 2D BN / SDS PAGE and proteins were analyzed by mass spectrometry. The resulting 2D gel and corresponding protein identification data were used to create a GelMap (https://gelmap.de/). The map includes 52 spots (circles) and overall 30 different complex I subunits with homologs in *A. thaliana*, as well as 3 additional putative diatom-specific complex I subunits. The molecular masses of standard proteins are given to the left of the protein gel. Numbers above the gel indicate estimated molecular masses of protein complexes on the first (native) gel dimension, numbers to the left indicate molecular masses of standard proteins on the second gel dimension (both in kDa). The GelMap is accessible at <https://www.gelmap.de/phaeo-ci/>. Note that the presented GelMap has been curated as follows: complex I proteins were only assigned to the spots for which they showed the highest iBAQ (intensity based absolute quantification) value, respectively; proteins detected within the spots that are known to form part of other protein complexes (e.g. photosystem I, photosystem II) were removed.

Supp. Figure 7


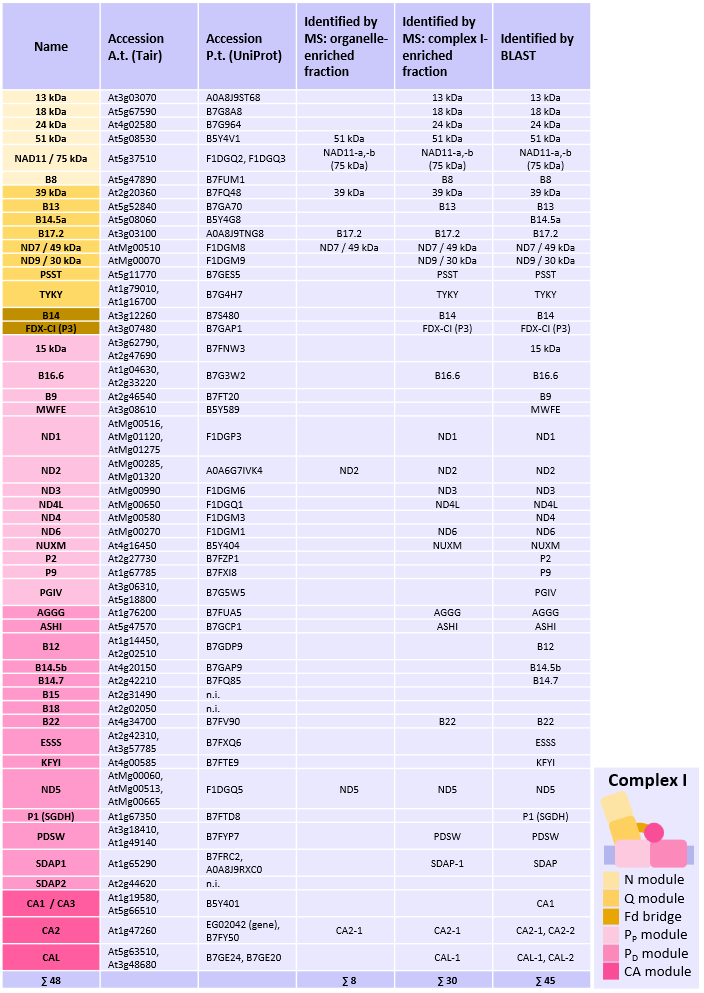


**Supplementary Figure 7: Complex I subunits of *A. thaliana* and their (putative) homologs in** ***P. tricornutum*.** The data included in this table are the basis for Fig. 8. Homologs of 45 out of 48 complex I subunits from *A. thaliana* were identified for *P. tricornutum* based on similarity searches using the genome sequence of *P. tricornutum*. Thirty of these were also identified by MS using our complex I-enriched fraction. Note that additional subunits were identified in the complex I-enriched fraction from *P. tricornutum*, which are absent in *A. thaliana*. One of these subunits, named unknown subunit 1, is undoubtedly a complex I subunit (Fig. 6, Supp Fig. 6 and Supp. Fig. 8). For further details see also Supp. Table 2.

Supp. Figure 8


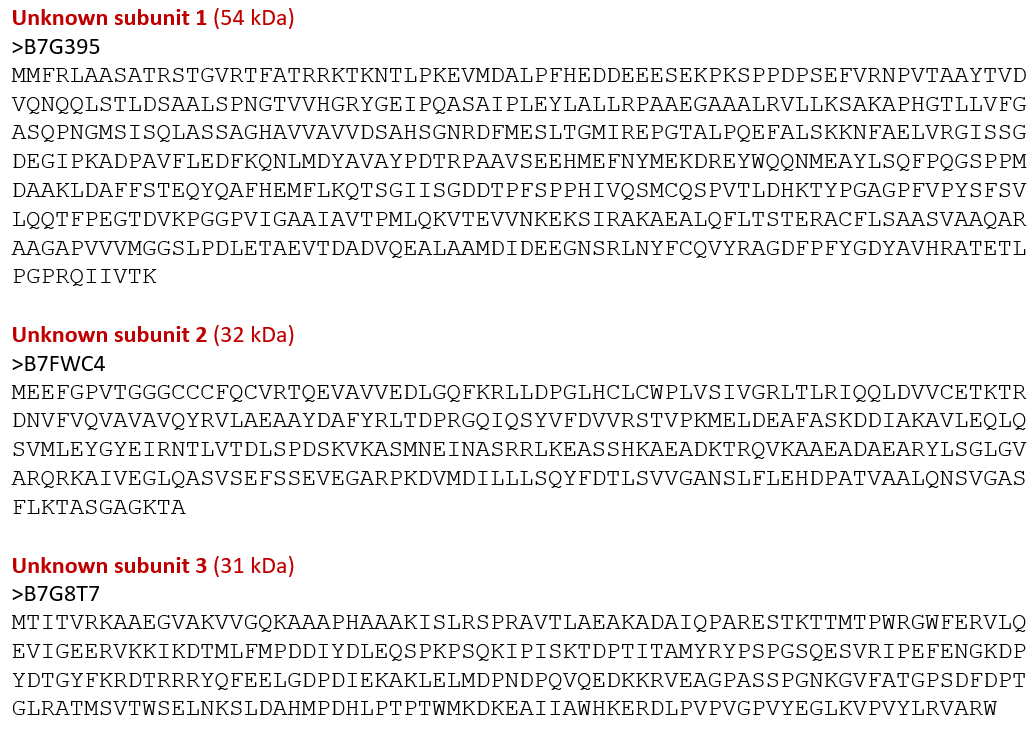


**Supplementary Figure 8: Additional proteins identified in the gel region of complex I (**[**https://www.gelmap.de/phaeo-ci/**](https://www.gelmap.de/phaeo-ci/)**,** Supp. Fig. 6**), which do not resemble known complex I subunits of other organisms.** Proteins as designated in the text, given in FASTA format with UniProt identifiers. Respective protein spots are labeled by asterisks in Fig. 6.

Supp. Figure 9


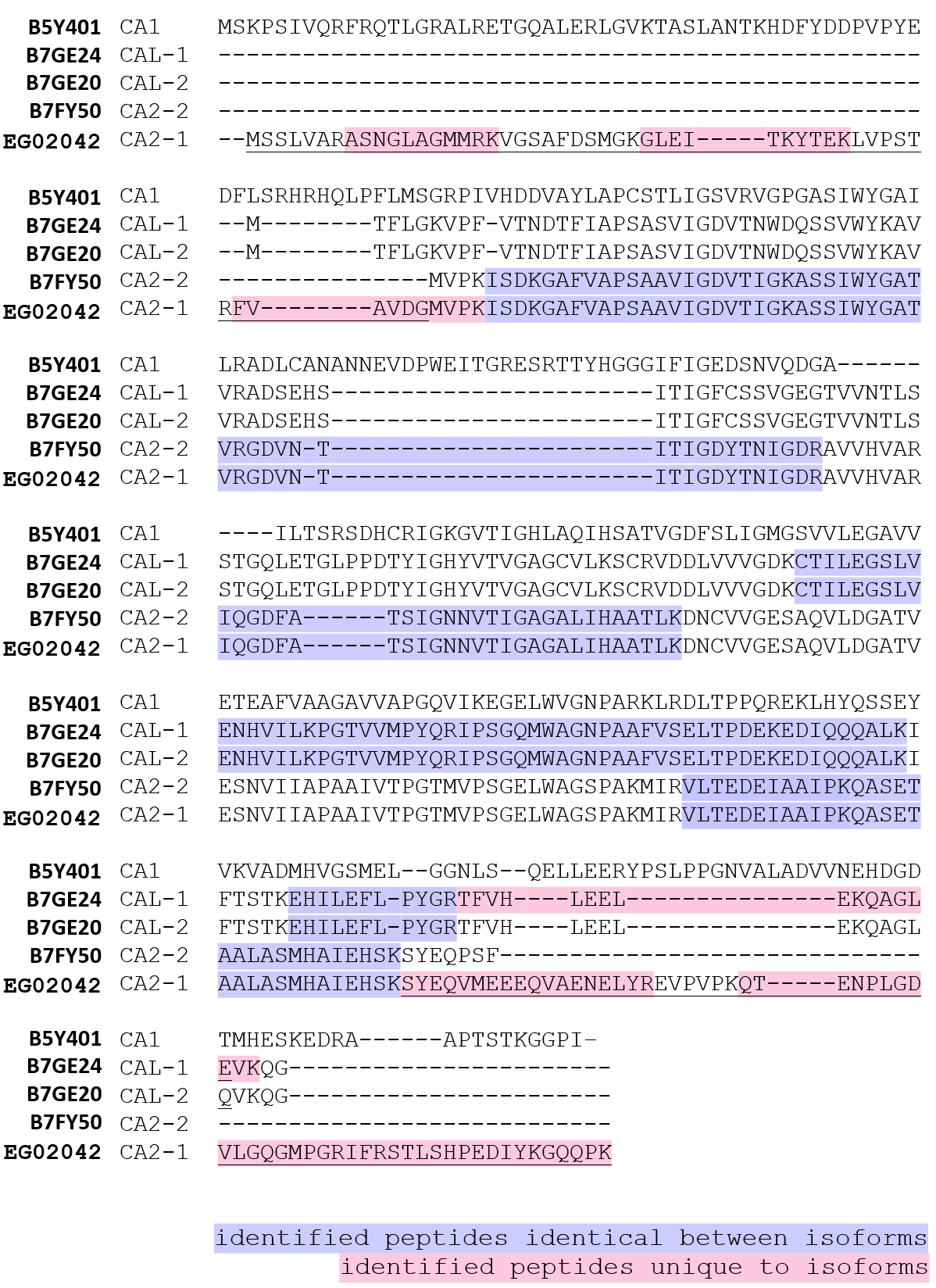


**Supplementary Figure 9: Multiple sequence alignment of *P. tricornutum* γ-type carbonic anhydrases and their coverage by detected peptides.** The γCA and γCAL subunits of *P. tricornutum* were aligned using Clustal Omega (<https://www.ebi.ac.uk> (Madeira *et al.*, 2024)). Identified peptides are highlighted in blue, unique peptides in pink. γCA2-1 and γCA2-2, as well as γCAL-1 and γCAL-2, are identical except for underlined amino acids. Note that no unique peptide was identified for γCA2-2 and γCAL-2, and no peptide was identified for γCA1. Accessions (& origins) of P.t. γCA(L)s are: CA1 = B5Y401 (UniProt), CAL-1 = B7GE24 (UniProt), CAL-2 = B7GE20 (UniProt), CA2-2 = B7FY50 (UniProt), CA2-1 = EG02042 (DiatOmicBase, www.diatomicsbase.bio.ens.psl.eu).

Supp. Figure 10


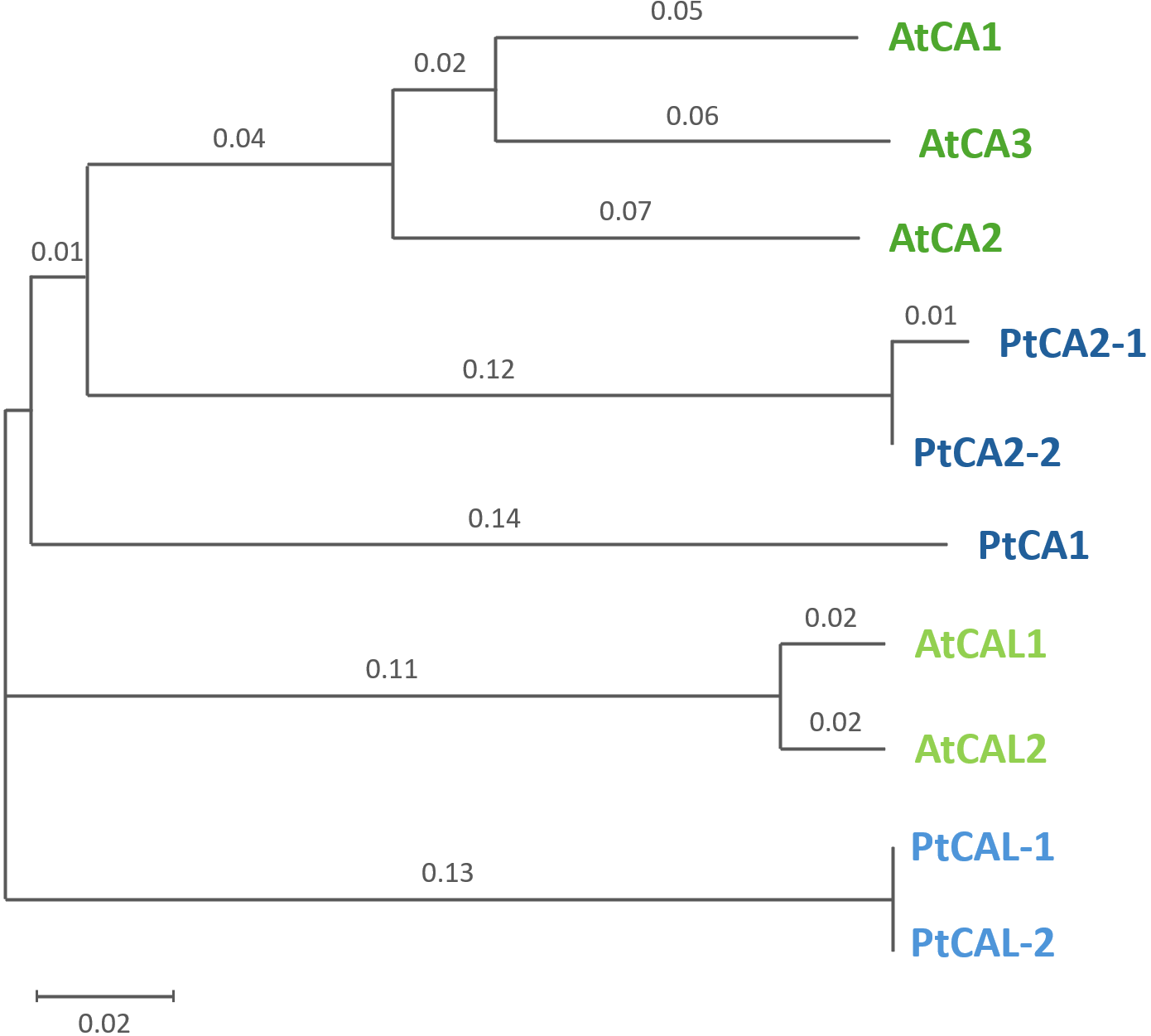


**Supplementary Figure 10:** **Sequence similarity tree of the amino acid sequences of the γCA and γCAL subunits of mitochondrial complex I encoded by the genomes of *A. thaliana* and *P. tricornutum*.** The tree was created with T Coffee (https://tcoffee.crg.eu/) using regular settings (Notredame *et al.*, 2000), and displayed with MEGA, version 12 (Kumar *et al.*, 2024). Accessions (& origins) of P.t. γCA(L)s are: PtCA1 = B5Y401 (UniProt), PtCAL-1 = B7GE24 (UniProt), PtCAL-2 = B7GE20 (UniProt), PtCA2-1 = EG02042 (DiatOmicBase, www.diatomicsbase.bio.ens.psl.eu), PtCA2-2 = B7FY50 (UniProt). Accessions for *A. thaliana* γCA/γCAL (all from TAIR, https://www.arabidopsis.org/) are: AtCA1 = At1g19580, AtCA2 = At1g47260, AtCA3 = At5g66510, AtCAL1 = At5g63510, AtCAL2 = At3g48680.

**Supplementary Table 1. MS data of the GelMap of the organelle-enriched fraction of *Phaeodactylum tricornutum*.**

**Supplementary Table 2**. **Complex I subunits of *Phaeodactylum tricornutum* (extended list of Supp. Fig. 7)**: List of *P. tricornutum* proteins compared with *A. thaliana* complex I subunits, with accession numbers, identification methods (BLAST, mass spectrometry, MS), theoretical molecular weight (MW) and isoelectric point (pI), as well as gene information (cDNA, gene ID, chromosome, and exon number).
